# Tracing microbial community across endophyte-to-saprotroph continuum of *Cinnamomum camphora* (L.) Presl leaves considering priority effect of endophyte on litter decomposition

**DOI:** 10.3389/fmicb.2024.1518569

**Published:** 2025-01-15

**Authors:** Jiamin Xiao, Zaihua He, Xingbing He, Yonghui Lin, Xiangshi Kong

**Affiliations:** ^1^College of Biology and Environmental Sciences, Jishou University, Jishou, China; ^2^College of Tourism and Management Engineering, Jishou University, Zhangjiajie, China

**Keywords:** community assembly, co-occurrence network, keystone taxa, leaf endophytes, microbial diversity

## Abstract

Endophytes typically coexist with plants in symbiosis and transition into the saprobic system as plant tissues senesce, participating in the decomposition process of litter. However, the dynamic changes of endophytic communities during this process and their role in litter decomposition remain unclear. This study tracked the microbial composition across the transition from live leaves to litter in *Cinnamomum camphora* (L.) Presl *(C. camphora)*, evaluating the contribution of endophytes to litter decomposition by examining microbial diversity, community assembly, and co-occurrence networks along the endophyte-to-saprotroph spectrum. The results revealed increasing bacterial diversity but stable fungal diversity, and the diversity of endogenous microbes is mirrored this in the saprophytic phase. Bacterial community assembly was characterized by deterministic processes during the symbiotic phase, shifted to stochastic processes during the saprophytic phase. In contrast, fungal community assembly was predominantly driven by stochastic processes throughout the continuum. Out of the 49 keystone taxa identified, only *Pseudorhodoplanes sinuspersici* demonstrated a significant positive correlation with community assembly. All identified bacterial keystone taxa during the saprophytic phase originated from endophytic sources, and around 80% of the fungal keystone taxa in the initial stages of decomposition were similarly endophytic in origin. Additionally, 60% of the dominant bacterial taxa and 28% of the dominant fungal taxa at the commencement of decomposition were of endophytic descent. This suggests that endogenous microbes possess the potential to evolve into both keystone and dominant taxa during the saprophytic phase. Endogenous keystone and dominant microbes both exhibited significant correlations with microbial network, indicating their substantial ecological presence in microbial community. Both endogenous keystone and dominant taxa exerted significant potential influences on litter decomposition. Overall, during the saprophytic phase, endophytes are likely to influence the assemblage of microbial communities, the network structure, and decomposition-related functions. Specifically, it appears that bacterial endophytes may possess a greater adaptability to the decomposition processes of leaf litter compared to their fungal counterparts.

## 1 Introduction

Living plants maintain a dynamic relationship with endophytic microorganisms, such as fungi and bacteria, which reside within plant tissues without causing harm (Vannier et al., 2018; Zhou et al., 2018). These endophytes engage in complex symbiotic interactions with their hosts, enhancing plant growth, nutrient uptake, and defense against herbivores and pathogens (Liu et al., 2019; Xiong et al., 2021). They also influence the broader food web and are essential for the plant’s ecological functions and environmental resilience (Bulgarelli et al., 2013; Vandenkoornhuyse et al., 2015). The complexity of endophyte-host interactions extends beyond symbiosis, necessitating a broader ecological view. Recent research has been exploring the “afterlife effects” of phytochemicals, challenging traditional views of symbiosis and saprophytism (LeRoy et al., 2011). Evidence is accumulating that symbiotic microbes, including foliar endophytes, play a key role in litter decomposition in terrestrial ecosystems, such as direct decompose the litter, the influence of nutrient cycling and the symbiosis and competition with bacteria (Hirose et al., 2013; Peršoh, 2013; Zhou et al., 2017). Osono (2006) found that approximately two-thirds of endophytic fungi also inhabit leaf litter, capable of colonizing fallen leaves and influencing microbial community dynamics through priority effects (Fukami, 2015; Cordovez et al., 2019). This insight bridges the previously distinct fields of endophytic and saprophytic research, recognizing them as interconnected components of a dynamic ecological spectrum, with endophytic microbes as transformational agents. The clear distinction between leaf symbiosis and saprophytic systems is fading (U’Ren and Arnold, 2016). However, the study of microbial community shifts along the endophyte-to-saprotroph continuum is still in its infancy, particularly regarding the community dynamics in the presence of endophytic microbes.

The microbial transition from endophytic to saprophytic lifestyles along the leaf endophyte-to-saprotroph continuum represents a critical ecological succession, highlighting distinct gradients across various habitats. Along this continuum, microbial communities shift in response to fluctuations in both the plant’s internal environment and the external saprophytic conditions (Zhou et al., 2018). Endophytes during the endogenous phase are largely derived from root translocation, seed transmission, or external sources (Compant et al., 2010; Vannier et al., 2018), with the plant’s internal milieu imposing significant selective pressures on endophytic microbiota assembly. Even if leaves senesce and the plant’s immune barriers weaken, some selective filtering still remains, influencing endophytic microbiota. In contrast, the saprophytic phase is characterized by reduced plant filtering, creating a more welcoming environment for microbial colonizers. The emergence of new niches and the availability of decomposing organic matter allow for a diversification of species, enriching the microbial community dynamically (Zhang et al., 2024). However, some endophytes exit the saprophytic system, either due to a lack of metabolic capabilities for saprophytic nutrition or limited litter decomposition abilities, leading to a decline in their diversity as decomposition progresses (Rodriguez and Redman, 1997). Against this backdrop, our *first hypothesis suggests* that across the endophyte-to-saprotroph continuum, there is an overall increase in microbial diversity, along with a decrease in the diversity of endophytic taxa that were abundant during the symbiotic phase, as the ecosystem transitions into the saprophytic phase.

Biodiversity’s emergence, encapsulated by “community assembly,” involves the intricate formation of species within ecological communities (Mittelbach and Schemske, 2015). In microbial ecosystems, this process is shaped by a complex array of spatio-temporal factors, with deterministic and stochastic processes being essential for understanding community structure and evolution (Graham et al., 2017; Vilmi et al., 2021; Wang et al., 2021). During the plant endophytic stage, microbial community composition is influenced by both the soil microorganism pool and the plant’s selective recruitment within the endosphere (Vannier et al., 2018). This selection involves plant defense mechanisms and signaling pathways, as well as the promotion of beneficial microbes through nutritional exclusion (Yamada et al., 2016; Kiers et al., 2011; Vandenkoornhuyse et al., 2015). This selective filtering acts as a barrier to exogenous microorganisms, suggesting that endophytic community assembly is largely deterministic. In contrast, the saprophytic phase, characterized by abundant resources, is dominated by stochastic processes reflecting the variability of colonization events (Chase, 2010; Dini-Andreote et al., 2014). Endogenous microbes transitioning to this phase can preferentially colonize litter and exploit decomposable organic matter, becoming pioneer decomposers. They are expected to significantly influence the succession and assembly of decomposer communities through priority effects and nutrient alterations (Yuan et al., 2011; Voøíškova and Baldrian, 2013; Wolfe and Ballhorn, 2020). Therefore, we propose the *second hypothesis* that while deterministic processes govern symbiotic phase assembly, stochastic processes primarily influence the saprophytic phase. Additionally, endophytes, particularly keystone species, are anticipated to substantially impact community assembly via priority effects.

In the dynamic mosaic of microbial community succession and assembly, microorganisms form intricate interaction networks that are vital for ecological function. Co-occurrence analysis, a cornerstone of community ecology, reveals keystone species within these networks, which have outsized impacts on ecosystem processes. Endophytes, preferentially colonizing litter, interact directly with other microbes and indirectly by modifying substrate availability, suggesting they may act as keystone species in microbial networks during decomposition. Research increasingly shows that microbial diversity and community composition are fundamental to litter decomposition (Pioli et al., 2020; Schroeter et al., 2022; Zhang et al., 2024). Slight structural variations can lead to substantial functional changes, especially among early-arriving or keystone species that significantly influence community assembly and decomposition dynamics (Lin et al., 2015; Fukami, 2015; Debray et al., 2022; Yang et al., 2022). Building on this, we propose the *third hypothesis* that endophytes, especially keystone species in microbial networks, may be integral to the efficiency of litter decomposition processes.

## 2 Materials and methods

### 2.1 Study sites and experimental design

This study was carried on artificial *C. camphora* forest at four different sites in Hunan, China. Within each site, a random selection of five representative plots, each measuring 10 m × 10 m, was conducted for the collection of living leaves and leaf litter. The sampling encompassed four developmental phases of living leaves: tender leaves (TL), mature leaves (ML), senescent leaves (SL), and newly fallen leaves (NL, classified as living due to chlorophyll presence)—and tracked the progression through four distinct stages of leaf litter decomposition: initial (Q1), early (Q2), middle (Q3), and late (Q4). This comprehensive sampling period extended from March 2020 to October 2021, with specific details on sampling dates and locations delineated in Supplementary Table 1.

For each living leaf phase, the harvested leaves were promptly transported to the laboratory on ice within a 24-h window. Following a rigorous surface sterilization protocol, the leaf samples were cryopreserved at −80°C for subsequent DNA extraction, which would facilitate a comprehensive analysis of the endophytic microbial community composition (Beckers et al., 2016). The sterilization process commenced with a 30-s rinse in sterile water, proceeded with a 2-min immersion in 70% ethanol, followed by a 5-min treatment in 2.5% NaClO solution with 0.1% Tween 80, and concluded with a final 30-s immersion in 70% ethanol. The leaves were then thoroughly rinsed five times with sterile deionized water and gently dried using sterile filter paper to prepare them for the extraction of genetic material.

During the apex of the leaf fall season (September–October), nylon litter traps, fashioned with a 1 mm mesh and spanning an area of 2 m × 2 m, were strategically positioned at a height of 0.5 m above the terrain within each designated plot to efficiently collect the most recently descended leaves. A portion of these gathered leaves were allocated as specimens for the NL stage analysis, while the remainder were utilized as substrate for the decomposition investigation. The study of leaf litter decomposition across various stages was facilitated by employing a litterbag methodology. Specifically, samples of NL, equivalent in mass to 5 g of material oven-dried at 50°C for a duration of 48 h, were secured within litterbags (constructed of 1 mm nylon mesh and measuring 20 cm × 20 cm) for the decomposition process. To reduce the potential influence of soil heterogeneity on the decomposition dynamics, all litterbags were arranged in close proximity to one another within a plot selected at random from the cohort of five representative plots.

At each site, a total of 40 litterbags were deployed within the litter layer for a comprehensive 1-year decomposition study. At intervals of 1, 3, 6, and 12 months, a random sample of 10 litterbags was collected from each site. Under sterile conditions, surface debris was meticulously removed, and three litterbags containing the litter samples were preserved at −80°C for DNA extraction and sequencing, aiming to dissect the community composition of saprophytic microbes. The remaining litter was utilized to assess mass loss, lignin and cellulose content, CO_2_ flux, and enzymatic activity. Concurrently, soil samples surrounding the litterbags were collected at each sampling period, after sieved through a 2 mm sieve to measure the soil’s physicochemical properties, providing a holistic understanding of the decomposition ecosystem.

### 2.2 Measured variables

Soil moisture was determined employing the gravimetric method, with samples dried in an oven at 105°C for 24 h. Soil pH was measured using a pH meter (PHS-2F, Leici, China) with a soil to water ratio of 1:2.5. The content of soil organic carbon (SOC) was assayed by the potassium dichromate-sulfuric acid oxidation method. Available nitrogen (AN) in the soil was evaluated using the alkali diffusion method. Available phosphorus (AP) was extracted with 0.5 M sodium bicarbonate (NaHCO_3_), followed by the addition of molybdenum to the filtrate, and then quantified using a UV-Vis spectrophotometer (UV2400, Sunny, China) at a wavelength of 880 nm. All protocols for the analysis of the soil’s physicochemical properties adhered to the procedures detailed by Bao (2023).

The mass loss of leaf litter in each litterbag was ascertained by calculating the differential in weight before and after on-site incubation at each sampling juncture, with the results presented as a percentage of the initial dry mass. The lignin and cellulose content within the leaf litter was quantified utilizing the acid detergent fiber (ADF) methodology in conjunction with sulfuric acid digestion, in accordance with the procedures detailed by Van Soest et al. (1991). The CO_2_ efflux emanating from the litter was gauged through the NaOH-HCl titration technique and articulated in terms of μmol g^–1^ dry litter per hour, adhering to the protocols delineated by Yang et al. (2020). The activities of cellulolytic enzymes—specifically exo-1,4-β-glucanase (C1) [EC 3.2.1.91], carboxymethyl cellulose (Cx) [EC 3.2.1.4], and β-glucosidase (BG) [EC 3.2.1.21]—were optically quantified at 540 nm via the DNS (3,5-dinitrosalicylic acid) assay. These enzyme activities were articulated in units equivalent to the micromoles of glucose released per gram of dry litter per hour (Tang et al., 2023). Furthermore, the activities of laccase [EC 1.10.3.2] and peroxidase [EC 1.11.1.7] were appraised conforming to the method explicated by Fioretto et al. (2000). These enzyme activities were ascertained as μmol of tolidine oxidized per minute, employing a molar extinction coefficient of 6,340 (Lin et al., 2015). Each assay was conducted in triplicate for every treatment to ensure analytical rigor and precision.

### 2.3 Gene amplification and sequence processing

Total DNA was extracted and purified from living leaves (surface sterilization according to above method) or litter samples using the Zymo Research Biomics DNA Microprep Kit (Cat#D4301, Zymo Research, Orange, CA, USA). The integrity of genomic DNA (gDNA) was assessed by 0.8% agarose gel electrophoresis, followed by nucleic acid concentration measurement using the TECAN F200 (PicoGreen dye method). GSS-16S and GSS-ITS analyses were performed on the TL, ML, SL, and NL samples, while 16S and ITS analyses were conducted on the Q1, Q2, Q3, and Q4 samples. The primer pairs 515F (5′-GTG YCA GCM GCC GCG GTA A-3′) and 806R (5′- GGA CTA CHV GGG TWT CTA AT-3′) were used to amplify the GSS-16S rRNA and 16S rRNA sequences (Caporaso et al., 2011). For the amplification of GSS-ITS rRNA and ITS rRNA sequences, the primer pairs ITS3 (5′-GAT GAA GAA CGY AGY RAA-3′) and ITS4 (5′-TCC TCG CTT ATT GAT ATG C-3′) were selected (Bellemain et al., 2010).

PCR reactions for endophytic and saprophytic microbial sequencing were performed in a 50 μl mixture containing 5 μl of 10× PCR Buffer for KOD-Plus-Neo, 2 μl of GSS Depletion Mix [used only for GSS-16S rRNA and GSS-ITS rRNA sequencing to inhibit chloroplast and mitochondrial DNA, as described by Zhang et al. (2019)], 5 μl of dNTPs (at 2 mM), 3 μl of 25 mM MgSO_4_, 1.5 μl of each primer, 1 μl of KOD-Plus-Neo (1 U/μl), 2 μl of template DNA, and 29/31 μl of H_2_O. The PCR reactions were carried out using the following program: an initial denaturation at 94°C for 1 min, followed by 25–30 cycles of denaturation at 94°C for 20 s, annealing at 54°C for 30 s, elongation at 72°C for 30 s, and a final extension at 72°C for 5 min, with a hold at 4°C. Each sample was amplified in triplicate, and the PCR products were pooled in equal quantities for subsequent library construction. The resulting PCR products were mixed with a 6× loading buffer, and the target fragments were visualized by electrophoresis on a 2% agarose gel. Samples that passed quality control were excised from the gel, purified using the Zymoclean Gel Recovery Kit (D4008), quantified with a Qubit 2.0 Fluorometer (Thermo Scientific), and pooled in equimolar concentrations. High-throughput sequencing of the library was conducted on an Illumina HiSeq platform (HiSeq PE250) using the HiSeq Rapid SBS Kit v2 (FC-402-4023, 500 Cycle). The raw reads have been deposited in the NCBI Sequence Read Archive under accession number PRJNA1066277.

### 2.4 Bioinformatic analyses

The double-terminal sequence obtained from the above sequencing process was assembled by FLASH (v1.2.11, Magoc and Salzberg, 2011), and the amplification sequence of each sample was distinguished and separated based on the tag sequence. After trimming, the forward and reverse reads with 25∼200c bp overlapping were combined to obtain longer sequences. Also, unqualified sequences were removed if they were too short or contained an undetermined base “N” by QIIME software (v1.8.0^1^). Following this, potential chimeric sequences were detected and removed by Uchime algorithm and gold database. Sequences were then clustered into operational taxonomic units (OTUs) at 97% sequence similarity using UPARSE (Edgar, 2013). UCLUST taxonomy and SILVA database (version 138^2^) were used to annotation analysis (Pruesse et al., 2007; Edgar, 2010). Representative sequences were multiple aligned by PyNAST (v1.2.2, Price et al., 2009) and phylogenetic tree was constructed with FastTree (v2.1.10, Caporaso et al., 2010).

### 2.5 Data analysis

Alpha diversity metrics, encompassing the Chao1 richness estimator, phylogenetic diversity (PD), and the Shannon and Simpson indices were computed for both bacterial and fungal communities. To gauge microbial beta diversity, principal coordinate analysis (PCoA) was conducted, utilizing the Bray–Curtis distances of normalized OTU data. The computation of both alpha and beta diversities was facilitated by the microeco package in R (Liu et al., 2021). Permutational multivariate analysis of variance (PERMANOVA) was conducted based on Bray–Curtis distance at the OTU level using the *adonis2* function of the vegan package in R (Oksanen et al., 2022). The Venn diagram analysis, facilitated by the VennDiagram package in R (Chen, 2022), rendered a visual representation of the shared microbial species across the various endophytic phases. Microbial Source Tracking, implemented through the FEAST package in R, was engaged to trace OTUs from the endophytic source to the saprophytic sink (Shenhav et al., 2019).

We performed network analysis to assess the complexity of the microbiome and to pinpoint potential keystone taxa across the endophyte-to-saprotroph continuum. To mitigate the impact of rare OTUs, we focused on those with a relative abundance greater than 0.005 (Wang et al., 2022). Networks were constructed based on robust correlations, utilizing Spearman’s rank correlation coefficients (|*r*| > 0.6) with *p* < 0.05, adjusted using the Benjamini–Hochberg procedure. These thresholds were applied to evaluate the interactions among microbial OTUs from various sites (Pan et al., 2024). The network analysis was facilitated by the igraph package (Csardi and Nepusz, 2006) and visualized employing Gephi.^3^ Network properties were determined utilizing the *net_properties. 4* function from the ggClusterNet package in R (Wen et al., 2022). Concurrently, we established a linear model between each network property and the developmental stages along the endophyte-to-saprotroph continuum, employing the *lm* function within R’s stats package. Using the *ZiPiPlot* function from the ggClusterNet package in R, we assessed both within-module connectivity (Zi) and among-modules connectivity (Pi) to identified the keystone taxa based on high within-module connectivity (Zi > 2.5) or high among-module connectivity (Pi > 0.62) (Guimerà and Amaral, 2005; Tang et al., 2023). Subsequently, endogenous keystone taxa were identified, and their relative abundances were quantified to explore potential correlations with other ecological variables. Furthermore, we extracted individual sample sub-networks utilizing the *subgraph* function from the igraph package in R, and subsequently calculated each sub-network to facilitate further analysis. Acknowledging the substantial role that dominant taxa have in ecological functions; we proceeded to conduct a comprehensive statistical analysis focused on the dominant taxa within the microbial community. The prevalent genera at each phase along the endophyte-to-saprotroph continuum were identified by selecting the top 10 dominant genera, which were designated as the dominant taxa. We then quantified the abundance of endogenous OTUs within these dominant genera for subsequent correlation analysis with other environmental variables. In addition, the count of endogenous OTUs within each genus was tallied to determine the number of dominant species, providing a metric for species richness at the genus level.

The microbial community assembly processes were assessed through null model analysis at various stages of the endophyte-to-saprotroph continuum (Stegen et al., 2013; Dini-Andreote et al., 2014). To elaborate, deviations in PD were quantified utilizing a null model-derived metric, specifically the β-nearest taxon index (βNTI). A | βNTI| value exceeding 2 signifies the predominance of deterministic processes, with a markedly lower (βNTI < −2, homogeneous selection) or higher (βNTI > 2, heterogeneous selection) phylogenetic turnover than what is expected under null scenarios. Conversely, a | βNTI| value less than 2 is indicative of stochastic processes taking the lead (Chase et al., 2011; Stegen et al., 2013).

Spearman’s rank correlation analysis was conducted to examine the correlations between the relative abundance of keystone endophytic and dominant bacterial and fungal taxa, decomposition functions, the proportion of stochastic versus deterministic processes, and environmental factors. A Holm’s adjusted *p*-value threshold of less than 0.05 was applied to determine statistically significant correlations, this analysis and visualization were used the “corrplot” package (Wei et al., 2024). Furthermore, a piecewise structural equation modeling approach was employed to elucidate the causal interplay among variables at each stage of the saprophytic continuum.

## 3 Results

### 3.1 Microbial community structure and diversity

A comprehensive analysis resulted in the clustering of a total of 21,577 bacterial and 5,025 fungal OTUs, defined by a 97% nucleotide identity threshold, derived from 3,086,644 high-quality bacterial reads and 3,166,280 fungal reads across 96 samples of living leaves and litter. The findings highlighted that, at the genus level, *Ralstonia* was the predominant genus throughout the entire spectrum of the endophyte-to-saprotroph continuum (Supplementary Figure 1A). *Rhizobacter* and *Clostridium sensu stricto 12* were identified as the predominant genera within living leaves exclusively, while the litter stages were characterized by the dominance of *Allorhizobium-Neorhizobium-Parahizobium-Rhizobium* (Supplementary Figure 1A). Within the fungal community, a clear differentiation in genus distribution was observed across the various stages of the endophyte-to-saprotroph continuum (Supplementary Figure 1B). *Zasmidium* emerged as the dominant genus in living leaves, only to experience a swift decline in leaf litter. *Mycosphaerelloides* maintained a significant presence throughout the different stages of living leaves, though its prevalence waned as the leaves decomposed. Genera such as *Toxicocladosporium*, *Sphaerulina*, *Camptophora*, and *Epicoccum* exerted a notable influence during one or two specific stages of leaf habitation but nearly disappeared from the litter. In a similar vein, *Calonectria*, *Helicodendron*, *Trechispora*, and *Paracylindrocarpon* were observed to rise to prominence in only one or two specific litter stages. At the phylum level of bacterial classification, Proteobacteria and Bacteroidetes were consistently dominant throughout the endophyte-to-saprotroph continuum, with Firmicutes asserting their dominance specifically within living leaves (Supplementary Figure 2A). Within the fungal kingdom, Ascomycota maintained a dominant position throughout the continuum, while Basidiomycota showed a progressive increase in prevalence along the decomposition sequence (Supplementary Figure 2B).

**FIGURE 1:**
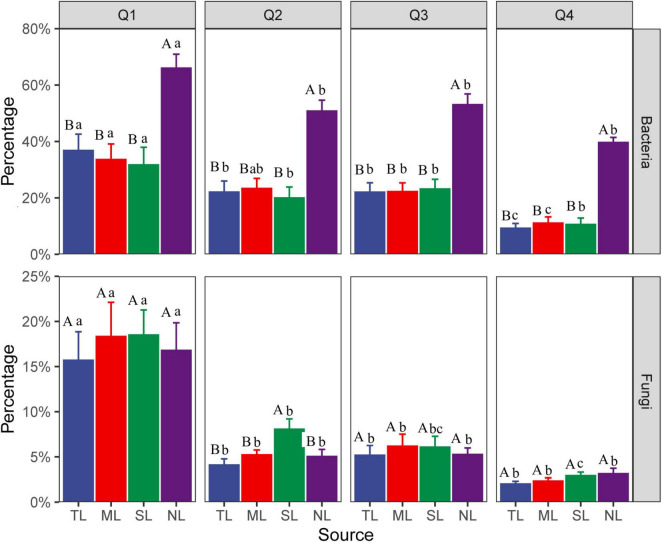
Microbial source tracking for the change in the number of endophytic OTUs transitioning from the source (endophytes) to the sink (saprophytes) in *C. camphora* leaves. Tender leaves (TL), mature leaves (ML), senescent leaves (SL), and newly fallen leaves (NL) are developmental phases of living leaves, and the Q1 (initial), Q2 (early), Q3 (middle), and Q4 (late) are the stages of leaf litter decomposition. Different uppercase letters indicate a significant difference between sources over the same decomposition stages with *p* < 0.05. The different lowercase letters indicate a significant difference between different decomposition stages over the same sources with *p* < 0.05.

The Venn diagram (Supplementary Figure 3) revealed that the bacterial and fungal communities at the endophytic phases shared approximately 5% and 10% of their species, respectively. The NL stage showcased a high degree of species exclusivity, particularly among bacteria, with 75.6% of species being unique to this stage. Microbial source tracking elucidated the transition of OTUs from the endophytic source to the saprophytic sink (Figure 1). Across the stages of litter decomposition, there was a noticeable decrease in the number of endophytic OTUs, with fungi showing a steeper decline than bacteria. For instance, by the late decomposition (Q4) stage, the number of bacterial OTUs had diminished to about 40% of the counts at the NL stage, while the representation of fungal OTUs had dwindled to below 5% across the same stages.

**FIGURE 2:**
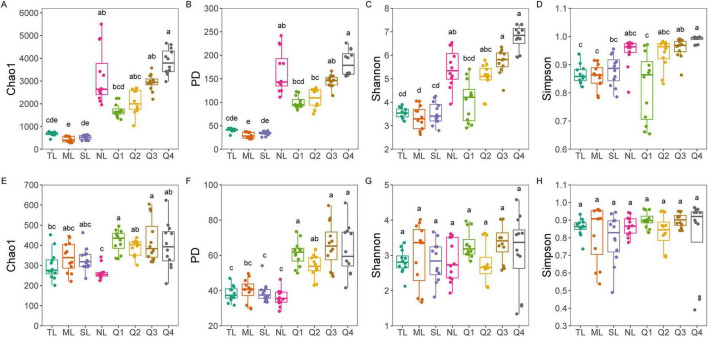
Bacterial **(A–D)** an fungal **(E–H)** diversity (including Chao1, PD, Shannon, and Simpson indices) along endophyte-to-saprotroph continuum of *C*. *camphora* leaves. Tender leaves (TL), mature leaves (ML), senescent leaves (SL), and newly fallen leaves (NL) are developmental phases of living leaves, and the Q1 (initial), Q2 (early), Q3 (middle), and Q4 (late) are the stages of leaf litter decomposition. Different lowercase letters indicate a significant difference with *p* < 0.05.

**FIGURE 3:**
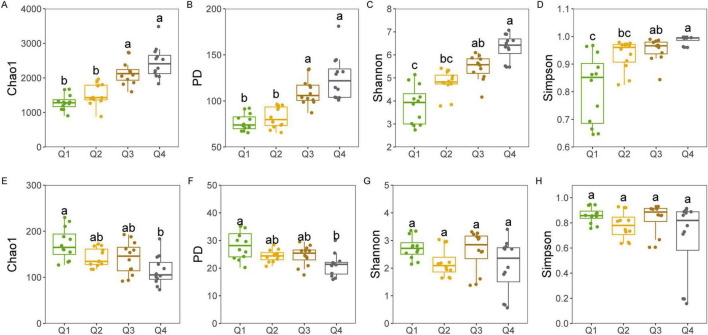
Endophytic bacterial **(A–D)** and fungal **(E–H)** alpha diversity indices at each litter decomposition stage of *C*. *camphora* leaves. Q1 (initial), Q2 (early), Q3 (middle), and Q4 (late) are the stages of leaf litter decomposition. Different lowercase letters indicate a significant difference with *p* < 0.05.

**FIGURE 4:**
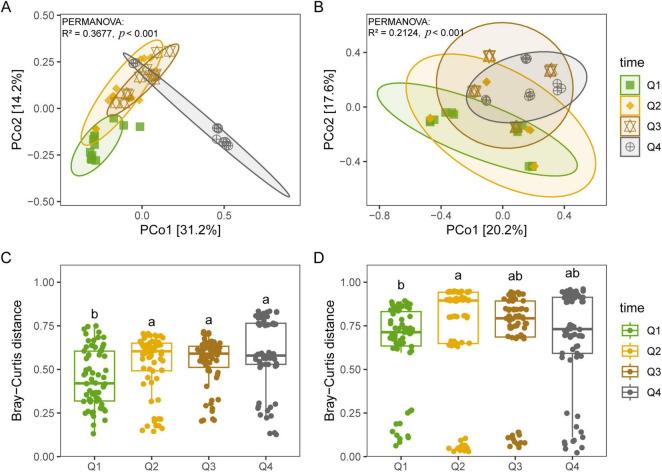
Principal coordinates analysis (PCoA) for endophytic bacterial **(A,C)** and fungal **(B,D)** communities at OTU level based on Bray–Curtis distance during litter decomposition stage of *C*. *camphora* leaves. Q1 (initial), Q2 (early), Q3 (middle), and Q4 (late) are the stages of leaf litter decomposition. Different lowercase letters indicate a significant difference with *p* < 0.05.

Bacterial diversity, encompassing the Chao1, PD, Shannon, and Simpson indices, revealed no significant differences among the first three stages of the leaf endophytic phase according to the Kruskal–Wallis test. However, a marked increase in diversity was observed at the NL stage (Figures 2A–D), followed by a sustained rise in bacterial diversity along the trajectory of litter decomposition. In contrast, fungal diversity did not exhibit a significant increase along the endophyte-to-saprotroph continuum for most indices, with the exception of the PD index (Figures 2E–H). Furthermore, we found that as litter decomposition progressed, all bacterial endophytic diversity indices continued to increase (Figures 3A–D). Conversely, the diversity indices of fungal endophytes showed only a marginal decline across the saprophytic stages, with no significant changes observed in most instances (Figures 3E–H). PCoA revealed that the endophytic bacterial (Figure 4A) and fungal (Figure 4B) communities at the OTU level from different litter stages formed discrete clusters in ordination space, with PERMANOVA indicating significant compositional differences (*p* < 0.01). Beta diversity assessments based on Bray–Curtis distance metrics suggested a lower rate of species turnover in the initial decomposition stage (Q1) for both endophytic bacteria (Figure 4C) and fungi (Figure 4D).

### 3.2 Microbial interactions relationship and the keystone taxa

**FIGURE 5:**
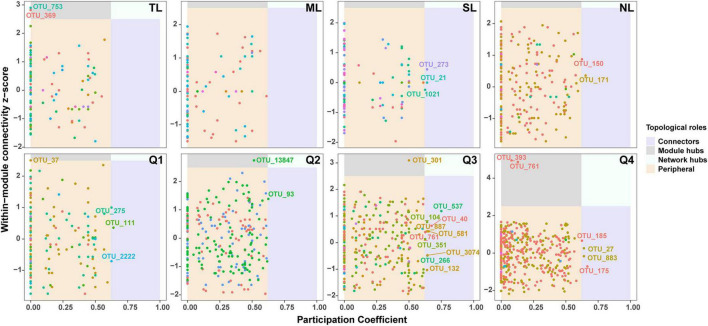
Zi-Pi plots showing distribution of OTUs based on their topological roles in bacterial networks during litter decomposition stage of *C*. *camphora* leaves. Tender leaves (TL), mature leaves (ML), senescent leaves (SL), and newly fallen leaves (NL) are developmental phases of living leaves, and the Q1 (initial), Q2 (early), Q3 (middle), and Q4 (late) are the stages of leaf litter decomposition.

**FIGURE 6:**
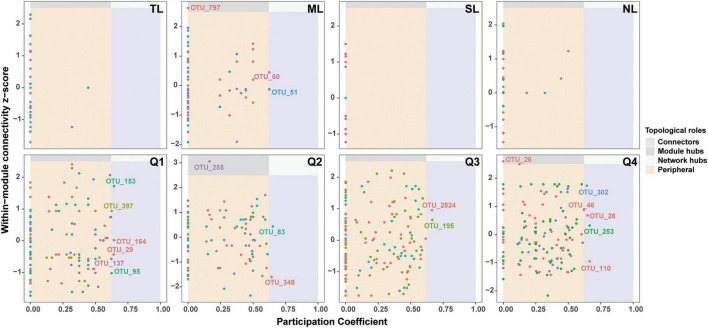
Zi-Pi plots showing distribution of OTUs based on their topological roles in fungal networks during litter decomposition stage of *C*. *camphora* leaves. Tender leaves (TL), mature leaves (ML), senescent leaves (SL), and newly fallen leaves (NL) are developmental phases of living leaves, and the Q1 (initial), Q2 (early), Q3 (middle), and Q4 (late) are the stages of leaf litter decomposition.

**FIGURE 7:**
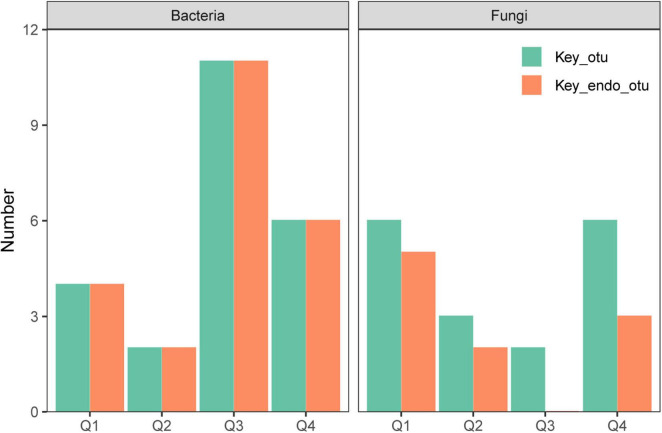
The proportion of endophytic keystone taxa to all keystone taxa of network for bacteria and fungi during litter decomposition stage of *C*. *camphora* leaves. Q1 (initial), Q2 (early), Q3 (middle), and Q4 (late) are the stages of leaf litter decomposition.

Supplementary Figure 4 illustrates the co-occurrence networks for bacterial and fungal species at each phase of the endophyte-to-saprotroph continuum. Linear modeling revealed significant temporal trends in network parameters for the majority of cases, signifying an escalation in network complexity as one moves along the continuum (Supplementary Figure 5 and Supplementary Table 2). Microbial network analysis, which emphasized within-module connectivity (Zi) and among-module connectivity (Pi), successfully identified 29 bacterial (Figure 5) and 20 fungal (Figure 6) keystone taxa. The majority of the bacterial keystone taxa were affiliated with the Proteobacteria and Bacteroidetes phyla, whereas the fungal keystone taxa were mainly represented by the Ascomycota division (Supplementary Tables 3, 4). Throughout the saprophytic stages, it was observed that all bacterial keystone taxa were of endophytic origin. In contrast, the fungal keystone taxa exhibited a variable proportion of endophytic species across the saprophytic stages, peaking at approximately 80% in Q1 stage and dropping to 0% in the middle decomposition stage (Q3) (Figure 7 and Supplementary Table 5). The ratio of dominant bacterial endophytic taxa to the total dominant bacterial taxa remained consistently around 60% across all saprophytic stages. Conversely, the representation of dominant fungal endophytic taxa was considerably lower, with a range that extended from 28% in Q1 stage to a mere 6% in Q4 stage (Supplementary Figure 6 and Supplementary Table 6). Certain bacterial endophytic keystone taxa, including OUT_37, OTU_266, OTU_40, and OTU_185, demonstrated notably strong positive or negative correlations with the majority of network parameters (Supplementary Figure 7A). Among fungi, a more limited subset—specifically OUT_397 and OUT_46—showed significant correlations with the majority of network parameters (Supplementary Figure 7B). In this study, a few dominant genera were found to include keystone taxa, as detailed in Supplementary Table 7. Furthermore, we attempted to analyze the interaction network structure after removing keystone species from the community. Our findings indicate obviously changes in the topological parameters of the microbial interaction network following the removal of these keystone species (Supplementary Table 8). Among the bacterial endophytic dominant taxa, *Acidovorax*, *Allorhizobium-Neorhizobium-Pararhizobium-Rhizobium*, *Pedobacter*, *Pir4 lineage*, *Promicromonospora*, *Pseudoxanthomonas*, *Ralstonia*, and *Streptomyces* exhibited significant positive or negative correlations with the majority of network parameters, as illustrated in Supplementary Figure 8A. In the fungal kingdom, the genera *Mycosphaerelloides* and *Zasmidium* were the only ones that displayed significant correlations with the majority of network parameters, as depicted in Supplementary Figure 8B.

### 3.3 Microbial community assembly processes

**FIGURE 8:**
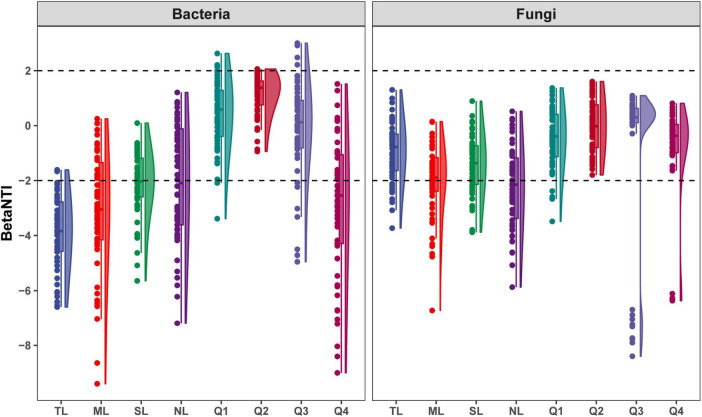
βNTI for bacterial and fungal community assembly along the endophyte-to-saprotroph continuum of *C*. *camphora* leaves. Tender leaves (TL), mature leaves (ML), senescent leaves (SL), and newly fallen leaves (NL) are developmental phases of living leaves, and the Q1 (initial), Q2 (early), Q3 (middle), and Q4 (late) are the stages of leaf litter decomposition.

Deterministic processes were found to be the predominant drivers of bacterial community assembly during the endophytic phases, while stochastic processes largely influenced assembly in most saprophytic stages, with a notable exception at the Q4 stage. Conversely, stochastic processes were the main determinants of fungal community assembly across nearly all stages, with a departure from this pattern observed at the NL stage (Figure 8 and Supplementary Figure 9). Among the endophytic keystone taxa, a significant positive correlation with the proportion of deterministic processes in community assembly was observed only for the bacterial OUT_266. In contrast, no substantial correlations were detected between the fungal endophytic keystone taxa and the assembly mechanisms of the community (Supplementary Figure 10). Within the bacterial endophytic dominant taxa, specific genera including *Allorhizobium-Neorhizobium-Parahizobium-Rhizobium*, *Bradyrhizobium*, *Enterobacter*, and *Luteibacter* showed notably positive or negative correlations with deterministic processes influencing community assembly. In the fungal realm, genera such as *Aspergillus*, *Beltrania*, and *Fusidium* also exhibited significant correlations with the deterministic processes at play in community assembly (Supplementary Figure 11).

### 3.4 Relationship of endophytes to decomposition function and environmental factors

Supplementary Figure 12 depicts the correlations between endophytic keystone microbes and decomposition functions, as well as environmental factors. The findings revealed that several bacterial endophytic keystone taxa, including OTU_37, OTU_266, OTU_93, OTU_887, OTU_132, OTU_581, OTU_883, and OTU_185, exhibited significant correlations, predominantly negative, with decomposition functions such as CO_2_ flux and the activity of decomposing enzymes. In contrast, fungal endophytic keystone taxa displayed a comparatively lower degree of correlation with the decomposition processes relative to their bacterial counterparts. A multitude of bacterial endophytic keystone taxa demonstrated significant correlations with the lignin and cellulose content within the litter. In contrast, none of the fungal endophytic keystone taxa exhibited significant correlations with the cellulose content. Among the array of environmental factors, pH had a higher prevalence of significant correlations, mostly negative, with bacterial endophytic keystone taxa such as OTU_111, OTU_275, OTU_13847, OTU_761, OTU_27, and OTU_185, when compared to other factors. Soil moisture also showed a considerable number of significant correlations, predominantly positive, with bacterial endophytic keystone taxa, including OTU_275, OTU_301, OTU_351, OTU_104, OTU_27, and OTU_185. For fungal endophytic keystone taxa, AN and SOC exhibited stronger correlations with these taxa than did other environmental factors.

Supplementary Figure 13 illustrates that, in the majority of cases, around half of the bacterial endophytic dominant taxa showed significant correlations with decomposition functions, including respiration rates and enzymatic activities. In contrast, a smaller proportion of fungal endophytic dominant taxa exhibited significant correlations with these decomposition processes. Furthermore, akin to keystone taxa, numerous endophytic dominant taxa also revealed strong correlations with environmental factors.

**FIGURE 9:**
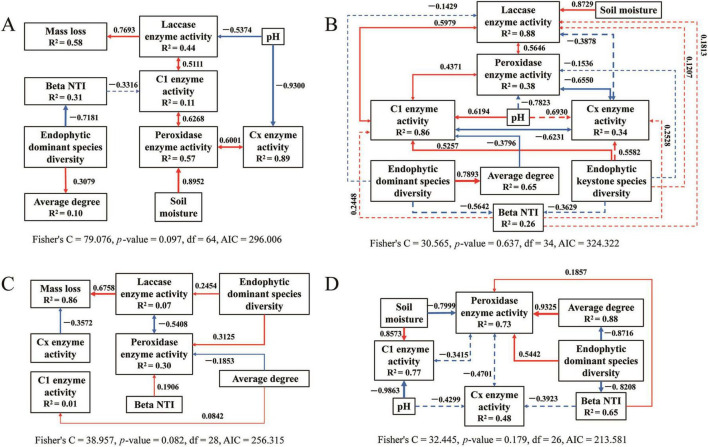
The piecewise structural equation model of analyzing the causal relationship between the variables for bacteria during litter decomposition stage of *C*. *camphora* leaves [panels **(A–D)** denote the sequential stages of leaf litter decomposition, corresponding to Q1 (initial), Q2 (early), Q3 (middle), and Q4 (late), respectively). Solid arrows indicate notable relationship, *p* < 0.05, dashed arrows *p* > 0.05, where the thickness of the arrow represents the strength of the relationship. Red and blue lines positive and negative relationships, respectively.

**FIGURE 10:**
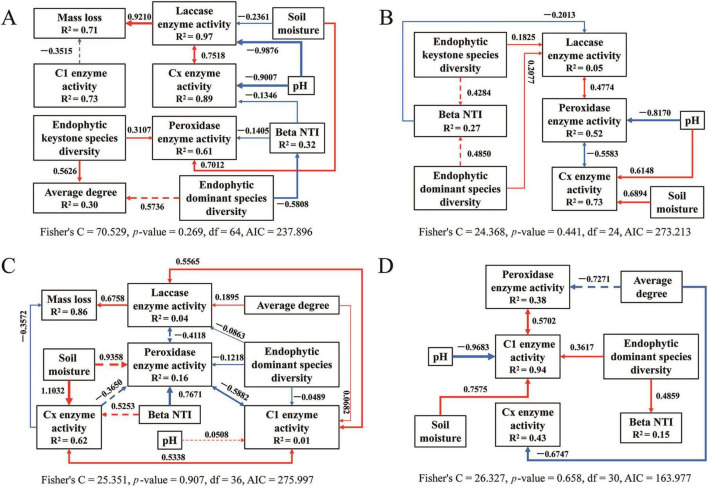
The piecewise structural equation model of analyzing the causal relationship between the variables for fungi during litter decomposition stage of *C*. *camphora* leaves [panels **(A–D)** denote the sequential stages of leaf litter decomposition, corresponding to Q1 (initial), Q2 (early), Q3 (middle), and Q4 (late), respectively]. Solid arrows indicate notable relationship, *p* < 0.05, dashed arrows *p* > 0.05, where the thickness of the arrow represents the strength of the relationship. Red and blue lines positive and negative relationships, respectively.

The structural equation model results for bacteria (Figure 9) and fungi (Figure 10) are detailed for each stage of the saprophytic continuum. Among the environmental factors considered, pH predominantly exerted a significant negative influence on the activity of degrading enzymes in most instances. In contrast, soil moisture typically exhibited a significant positive effect on enzymatic degradation. The presence of keystone endophytic bacterial and fungal species was observed to impact the activity of degrading enzymes or the complexity of microbial networks at specific stages of saprophytism. Moreover, the count of dominant endophytic species substantially influenced degrading enzymes, microbial network structure, and community assembly in the majority of cases.

## 4 Discussion

### 4.1 Changes in microbial diversity

Endophytes residing within living leaves have been extensively characterized, with specific species acknowledged for their role in facilitating litter decomposition (Schlegel et al., 2016; Zhou et al., 2017). Nonetheless, the dynamics of diversity and species composition as endophytes transition along the continuum to become saprotrophs remain largely uncharted territory (U’Ren and Arnold, 2016). This study delves into the variations in microbial diversity along the endophyte-to-saprotroph continuum. A marked increase in bacterial diversity was observed during the symbiotic phase, particularly in NL, which may indicate a substantial decline in the defensive mechanisms of the leaves as they undergo senescence. This surge could be attributed to opportunistic bacteria rapidly colonizing the leaves through physical invasion, as proposed by Saleem (2021) and Trivedi et al. (2022). In contrast, there was a noticeable decrease in bacterial diversity as the process transitioned from the NL phase to the initial stages of litter decomposition, possibly due to the inability of many endogenous bacteria to adapt to the saprophytic decay process, as noted by Osono (2006). Diversity later rebounded as decomposition progressed, likely due to the colonization by local species and the availability of readily accessible nutrients, such as secondary sugars released during decomposition (Cotrufo et al., 2013; Peršoh, 2013). Contrary to expectations, fungal diversity did not exhibit a significant increase along the endophyte-to-saprotroph continuum. Despite fungi’s reputed ability to invade plant tissues with ease, fungal diversity remained stable during the symbiotic phase, even as plant defenses weakened in the newly fallen leaf stage. Venn plot analysis indicated that only 25% of fungi were exclusive to this stage, compared to 75% of bacteria, suggesting a lower rate of exogenous infection for fungi. Nevertheless, this exclusivity implies that fungi can still effectively invade living leaves. The stable fungal diversity may be due to the saturation of ecological niches within the leaf endophytic community (Bills et al., 2012). As decomposition proceeds, some endogenous bacteria fail to adapt to the saprophytic phase, and the vacated niches are quickly filled by saprophytic fungi from the microbial pool. The high lignin content in *C*. *camphora* litter (approximately 45%) supports a stable turnover of “classic” decomposers, particularly the fungal community, thus maintaining relatively stable fungal diversity (Cornwell et al., 2009; Treseder and Lennon, 2015). This hypothesis is supported by the observed changes in βNTI values during the assembly of the fungal community.

As endophytic microorganisms transition into the saprophytic phase, they constitute a notable segment of the decomposer community, with certain endophytic fungi persisting in highly decomposed litter owing to their ligninolytic capabilities (Peršoh, 2013). Microbial source tracking disclosed a gradual diminution of endophytic bacteria from symbiotic stages during the decomposition process, aligning with the understanding that these microbes generally possess limited abilities for decomposing litter (Unterseher et al., 2013). Contrary to expectations, however, the diversity of endophytic bacteria increased during decomposition, implying a contribution from both the initial endogenous bacteria and the local species pool. In this study, the litter initially harbored endogenous bacteria from the newly fallen leaf stage, suggesting that previous assertions regarding the decline of endophytes during decomposition might have been biased by comparisons with earlier stages, without accounting for the local microbial pool’s input or the potential underestimation of exclusive endophytic species (Wolfe and Ballhorn, 2020). In contrast to bacteria, endogenous fungal diversity exhibited only a marginal decline across decomposition stages, despite the influx from external endophytes. This stability is likely a reflection of the low-quality litter substrate and is in concordance with patterns observed within broader saprophytic fungal communities. Furthermore, the observed weak correlation between keystone endophytic fungi and decomposition functionality bolsters this pattern, indicating that while endophytic fungi are present, their role in decomposition may not be as pronounced as that of their bacterial counterparts.

### 4.2 Relationship of endophyte to microbial community assembly

Microbial diversity at a given moment within a specific habitat represents a snapshot of the species composition within microbial communities, sculpted by the dynamic interactions among community members over time—a process quintessential to community assembly (Jiao and Lu, 2020). Our research indicates that bacterial community assembly is predominantly deterministic during the symbiotic phase, shifting to a stochastic process during the saprophytic phase. This is evidenced by an increasing trend in βNTI values along the symbiotic-to-saprophytic continuum, supporting our hypothesis that the filtration systems within *C*. *camphora* leaves impose significant selective pressure on microbial communities, impacting assembly even during the invasion by exogenous species in the newly fallen leaf stage. In contrast to bacteria, fungal communities are primarily driven by stochastic processes throughout the endophyte-to-saprotroph transition, which partially contradicts our initial hypothesis. This suggests that fungal communities may be less influenced by plant tissue filtration systems during the symbiotic phase, potentially due to a stable mutualistic relationship with *C*. *camphora* within the local ecosystem. This relationship likely enables them to effectively navigate plant defenses and signaling recognition systems (Lu et al., 2021; Akram et al., 2023). Furthermore, symbiotic fungal diversity is significantly lower compared to bacterial diversity, with Cordovez et al. (2019) proposing that stochastic changes play a pivotal role in the assembly of such low-diversity endogenous microbial communities.

During the saprophytic phase, the assembly of the fungal community is predominantly stochastic, enabling fungi to exploit the high lignin content in *C*. *camphora* litter with relative stability, without experiencing intense selective pressure on community turnover. The priority effect of endophytic microbes is posited to significantly influence community composition and interactions throughout the saprophytic process (Andersson et al., 2014; Bar-Massada and Belmaker, 2017). It is inferred that endophytes, particularly keystone species with substantial associations within the microbial network, play a pivotal role in community assembly. However, among the 49 keystone species identified, only the bacterial species *Pseudorhodoplanes sinuspersici* (OUT_266) demonstrated a significantly positive association with community assembly. Considering the ecological significance of dominance, we examined its relationship with community assembly. The findings indicated that both endophytic fungi and bacteria exhibited multiple dominant taxa that were closely associated with community assembly. Structural equation modeling analysis corroborated these results, suggesting that dominant endophytic taxa may exert a more substantial impact on community assembly due to their numerical superiority over keystone taxa. This insight underscores the importance of considering both keystone and dominant species when assessing the ecological roles of endophytes in the decomposition process.

### 4.3 Relationship of endophyte to microbial network

During the assembly of microbial communities, intricate network interactions are forged. Keystone species, known for their pivotal roles in maintaining ecological functions, interact extensively within these networks (Blanchet et al., 2020). Endophytes, leveraging the priority effect, establish an early presence in decomposing litter, positioning them as potential keystone species within microbial networks (He et al., 2017; Meng et al., 2023). Our study identified all keystone bacterial species during the litter decomposition process as being of endogenous origin. Similarly, at the outset of decomposition, approximately 80% of keystone fungal species were also indigenous. These results substantiate our hypothesis. Furthermore, up to 60% of dominant bacteria and 28% of dominant fungi during the saprophytic phase were found to be endogenous, which is in line with our expectations. Among these endophytic microbes, some keystone and dominant taxa exhibited a close correlation with the microbial network, indicating that endophytes have a significant impact on both network structure and community composition as they enter the decomposition process. Notably, endophytic bacteria displayed a more pronounced presence in network associations compared to endophytic fungi. This dominance may stem from their higher diversity and more efficient exploitation of soluble organic matter. Source tracking analysis further revealed that while 40% of endogenous bacteria persisted to the final decomposition stage (Q4), the presence of fungi dwindled to less than 5%. This endurance provides endogenous bacteria with a competitive advantage, enabling them to predominate in the composition and network structure of the microbial community.

Environmental factors also influence the role of endophytes within the network. pH and soil moisture likely significantly impacted key endogenous bacteria, while AN and SOC were closely associated with the performance of key endogenous fungi. This may be due to the fact that the optimal growth pH range for bacteria is typically narrower than that for fungi (Rousk et al., 2010). Meanwhile, we found that compared to phosphorus, AN may serve as a significant limiting factor, particularly for endophytic fungi. Meanwhile, Moore et al. (2020) reported that fungal decomposition of soil organic matter depends on soil nitrogen (N) availability, and the fungal biomass was reduced by N deposition.

### 4.4 Relationship of endophyte to litter decomposition

Endogenous microbes exert a substantial influence on the composition and structure of microbial communities, potentially shaping their ecological functions as well. Keystone endogenous bacteria, such as *Escherichia coli* TOP291 (OUT_37) and *P. sinuspersici* (OUT_266), are strongly correlated with mass loss and respiration, suggesting their pivotal role in litter decomposition. This impact is hypothesized to be mediated by their enhancement of enzymatic activities, including those of laccase, peroxidase, and C1 (*p* < 0.05, Supplementary Figure 12). This suggests that key endogenous bacteria possess the capability to degrade recalcitrant organic matter such as lignin and cellulose. The findings of Tirandaz et al. (2015) support our hypothesis which demonstrating that *P. sinuspersici* utilizes only complex carbon sources and pyruvate as the sole carbon source. Conversely, the majority of keystone endogenous fungi, with few exceptions such as *Dissoconium proteae* (OUT_397) and *Diaporthe amygdali* (OUT_348), exhibit significance but slight correlation with mass loss or respiration (*p* < 0.05, Supplementary Figure 12). This could be attributed to the higher turnover rate and metabolic activity of bacteria compared to fungi (Huang et al., 2021). Dominant endogenous bacteria also significantly affect enzyme activities and decomposition, while the influence of dominant endogenous fungi is comparatively weaker. These insights underscore the critical importance of bacterial activity in litter decomposition, shifting the focus from fungi, which have been conventionally considered the primary decomposers (Ye et al., 2024; Xu et al., 2013; Tláskal et al., 2016). Structural equation model analysis also indicates that both keystone and dominant endogenous microbes have stage-specific effects on decomposition functions. This implies that historical microbial colonization events, significantly influence decomposition processes (Dickie et al., 2012; Andersson et al., 2014).

By tracking the microbial community shifts along the endophyte-to-saprotroph continuum and analyzing the priority effects of endophytes on litter decomposition processes, we can identify keystone endophytic taxa that play a crucial role in the decomposition of litter. Utilizing these identified keystone taxa as an important selection for microbial manipulation in artificial forest ecosystems can help regulate the rate of litter decomposition, promote soil nutrient cycling, and thereby contribute to the ecological conservation and management of artificial forests.

## 5 Conclusion

The ecological transition of endophytic microorganisms from living leaves to litter involves a shift in nutritional strategies with implications that are not fully understood. These microbes, colonizing decaying litter through a priority effect, play a significant role in decomposition processes. Microbial tracing analysis indicates a decline in endophytic diversity during advanced decomposition stages, yet endophytic bacteria and fungi in litter maintain stable diversity, suggesting a robust species pool capable of replenishing decaying litter communities. The mechanisms influencing endophytic microbial diversity during the saprotrophic phase require further investigation, particularly regarding how the species pool affects community composition and responds to litter nutrient composition. Such insights are crucial for understanding the broader ecological roles of endophytes.

Endophytes are strongly correlated with decomposition functions at both the strain and community levels, indicating their potential to influence decomposition post-senescence. Interestingly, compared to endophytic fungi, endophytic bacteria appear to be more adapted to the decomposition process of litter, prompting us to reassess traditional viewpoints and suggesting that future research should focus on the role of endophytic bacteria in the decomposition process.

## Data Availability

The datasets presented in this study can be found in online repositories. The names of the repository/repositories and accession number(s) can be found in this article/Supplementary material.

## Footnotes

1
http://qiime.org/

2
https://www.arb-silva.de/

3
http://gephi.github.io/
