# Supplementary material for: Tracing microbial community across endophyte-to-saprotroph continuum of Cinnamomum camphora (L.) Presl leaves considering priority effect of endophyte on litter decomposition
Source: Front Microbiol. 2025 Jan 15;15:1518569. doi: 10.3389/fmicb.2024.1518569 (PMC11774851; doi:10.3389/fmicb.2024.1518569)
Supplement: Supplementary file 1 [file Data_Sheet_1.pdf]

Tracing microbial community across endophyte-to-saprotroph continuum of *Cinnamomum camphora* (L.)Presl leaves considering priority effect of endophyte on litter decomposition

Jiamin Xiao[1], Zaihua He[1], Xingbing He[1], Yonghui Lin[1,*], Xiangshi Kong[2]

[1]College of Biology and Environmental Sciences, Jishou University, Jishou 416000, China

[2]College of Tourism and Management Engineering, Jishou University, Zhangjiajie 427000, China

[*] **Corresponding author:**

Yonghui Lin

E–mail: **linyonghui@jsu.edu.cn**

Address: College of Biology and Environmental Sciences, Jishou University, Jishou, Hunan, 416000, China.

The captions of Supplementary Figure

Figure S1 Composition of bacterial (A) and fungal (B) taxa at the genus level along the endophyte-to-saprotroph continuum of *C. camphora* leaves. TL (tender leaves), ML (mature leaves), SL (senescent leaves) and NL (newly-fallen leaves) are developmental phases of living leaves, and the Q1 (initial), Q2 (early), Q3 (middle) and Q4 (late) are the stages of leaf litter decomposition.

Figure S2 Composition of bacterial (A) and fungal (B) taxa at the phylum level along the endophyte-to-saprotroph continuum of *C. camphora* leaves. TL (tender leaves), ML (mature leaves), SL (senescent leaves) and NL (newly-fallen leaves) are developmental phases of living leaves, and the Q1 (initial), Q2 (early), Q3 (middle) and Q4 (late) are the stages of leaf litter decomposition.

Figure S3 Venn diagram of endophytic bacterial (A) and fungal (B) communities in living leaves of *C. camphora*. TL (tender leaves), ML (mature leaves), SL (senescent leaves) and NL (newly-fallen leaves) are developmental phases of living leaves.

Figure S4 The co-occurrence networks for bacteria and fungi at each stage along the endophyte-to-saprotroph continuum of *C. camphora* leaves. TL (tender leaves), ML (mature leaves), SL (senescent leaves) and NL (newly-fallen leaves) are developmental phases of living leaves, and the Q1 (initial), Q2 (early), Q3 (middle) and Q4 (late) are the stages of leaf litter decomposition.

Figure S5 Linear regression of network parameters over time along the endophyte-to-saprotroph continuum of *C. camphora* leaves. TL (tender leaves), ML (mature leaves), SL (senescent leaves) and NL (newly-fallen leaves) are

developmental phases of living leaves, and the Q1 (initial), Q2 (early), Q3 (middle) and Q4 (late) are the stages of leaf litter decomposition.

Figure S6 The proportion of dominant endophytic taxa to all dominant taxa of network for bacteria and fungi during leaf litter decomposition of *C. camphora*. Q1 (initial), Q2 (early), Q3 (middle) and Q4 (late) are the stages of leaf litter decomposition.

Figure S7 The correlations between bacterial (A) and fungal (B) endophytic keystone taxa and network parameters during leaf litter decomposition of *C. camphora*. ("***" indicates significant difference with p <0.001; "**" indicates significant difference with p <0.01; "*" indicates significant difference with p <0.05.)

Figure S8 The correlations between bacterial (A) and fungal (B) endophytic dominant taxa and network parameters during leaf litter decomposition of *C. camphora*. ("***" indicates significant difference with p <0.001; "**" indicates significant difference with p <0.01; "*" indicates significant difference with p <0.05.)

Figure S9 The percentage of stochastic and deterministic processes during bacterial and fungal community assembly along the endophyte-to-saprotroph continuum of *C. camphora* leaves. TL (tender leaves), ML (mature leaves), SL (senescent leaves) and NL (newly-fallen leaves) are developmental phases of living leaves,

and the Q1 (initial), Q2 (early), Q3 (middle) and Q4 (late) are the stages of leaf litter decomposition.

Figure S10 The correlations between bacterial or fungal endophytic keystone taxa and community assembly during leaf litter decomposition of *C. camphora*. (“*” indicates significant difference with $p < 0.05$.)

Figure S11 The correlations between bacterial or fungal endophytic dominant taxa and community assembly during leaf litter decomposition of *C. camphora*. (“***” indicates significant difference with $p < 0.001$; “**” indicates significant difference with $p < 0.01$; “*” indicates significant difference with $p < 0.05$.)

Figure S12 The correlations of endophytes to decomposition functions and environmental factors during leaf litter decomposition of *C. camphora*. (“***” indicates significant difference with $p < 0.001$; “**” indicates significant difference with $p < 0.01$; “*” indicates significant difference with $p < 0.05$.)

Figure S13 The correlations of dominant taxa to decomposition functions and environmental factors during leaf litter decomposition of *C. camphora*. (“***” indicates significant difference with $p < 0.001$; “**” indicates

significant difference with $p < 0.01$; “*” indicates significant difference with $p < 0.05$.)

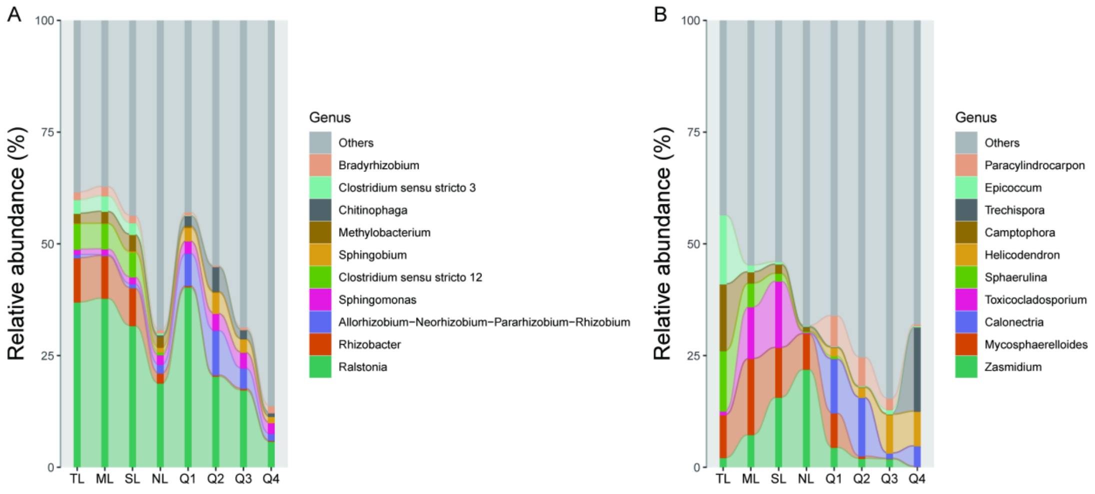
A
B
Relative abundance (%)
100
75
50
25
0
TL
ML
SL
NL
Q1
Q2
Q3
Q4
Genus
Others
Bradyrhizobium
Clostridium sensu stricto 3
Chitinophaga
Methylobacterium
Sphingobium
Clostridium sensu stricto 12
Sphingomonas
Allorhizobium−Neorhizobium−Pararhizobium−Rhizobium
Rhizobacter
Ralstonia
Paracylindrocarpon
Epicoccum
Trechispora
Camptophora
Helicodendron
Sphaerulina
Toxicocladosporium
Calonectria
Mycosphaerelloides
Zasmidium

Figure S1

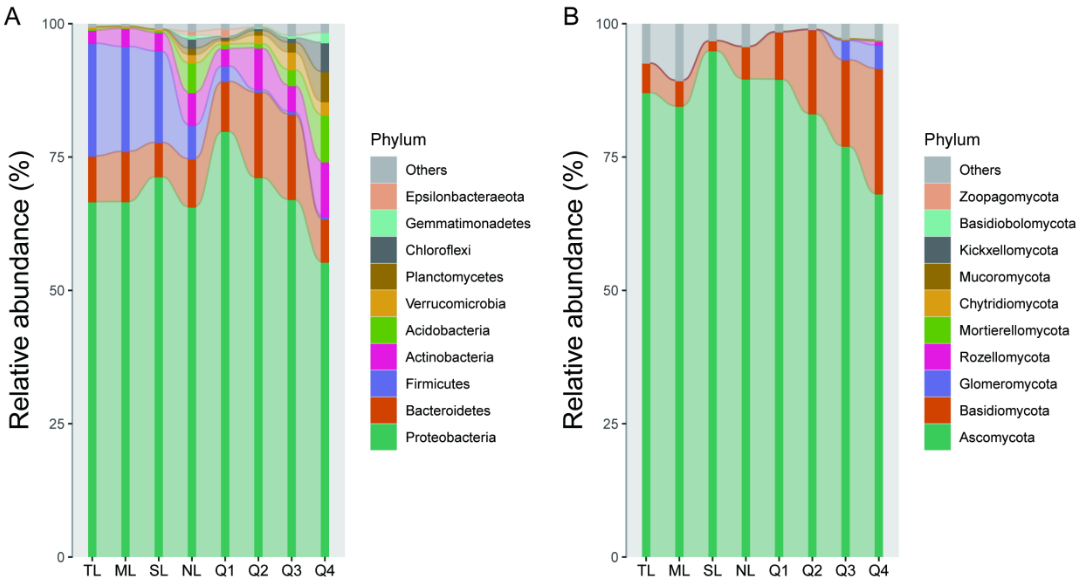
A
B
Relative abundance (%)
100
75
50
25
0
TL
ML
SL
NL
Q1
Q2
Q3
Q4
Phylum
Others
Epsilonbacteraeota
Gemmatimonadetes
Chloroflexi
Planctomycetes
Verrucomicrobia
Acidobacteria
Actinobacteria
Firmicutes
Bacteroidetes
Proteobacteria
Phylum
Others
Zoopagomycota
Basidiobolomycota
Kickxellomycota
Mucoromycota
Chytridiomycota
Mortierellomycota
Rozellomycota
Glomeromycota
Basidiomycota
Ascomycota

Figure S2

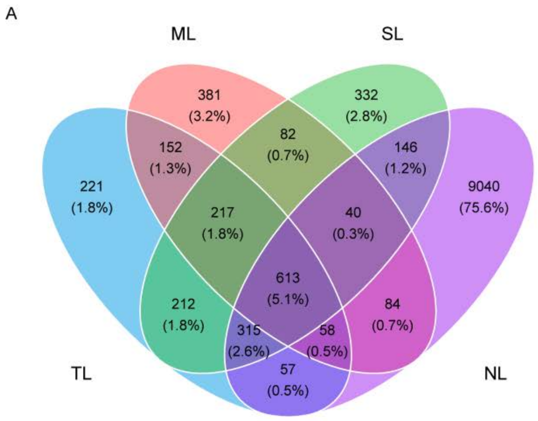
A
ML
SL
381
(3.2%)
332
(2.8%)
152
(1.3%)
82
(0.7%)
146
(1.2%)
221
(1.8%)
217
(1.8%)
40
(0.3%)
9040
(75.6%)
613
(5.1%)
212
(1.8%)
84
(0.7%)
315
(2.6%)
58
(0.5%)
57
(0.5%)
TL
NL
B
ML
SL
196
(9.5%)
218
(10.6%)
102
(4.9%)
130
(6.3%)
46
(2.2%)
183
(8.9%)
244
(11.8%)
54
(2.6%)
523
(25.4%)
211
(10.2%)
75
(3.6%)
26
(1.3%)
21
(1.0%)
21
(1.0%)
12
(0.6%)
TL
NL

Figure S3

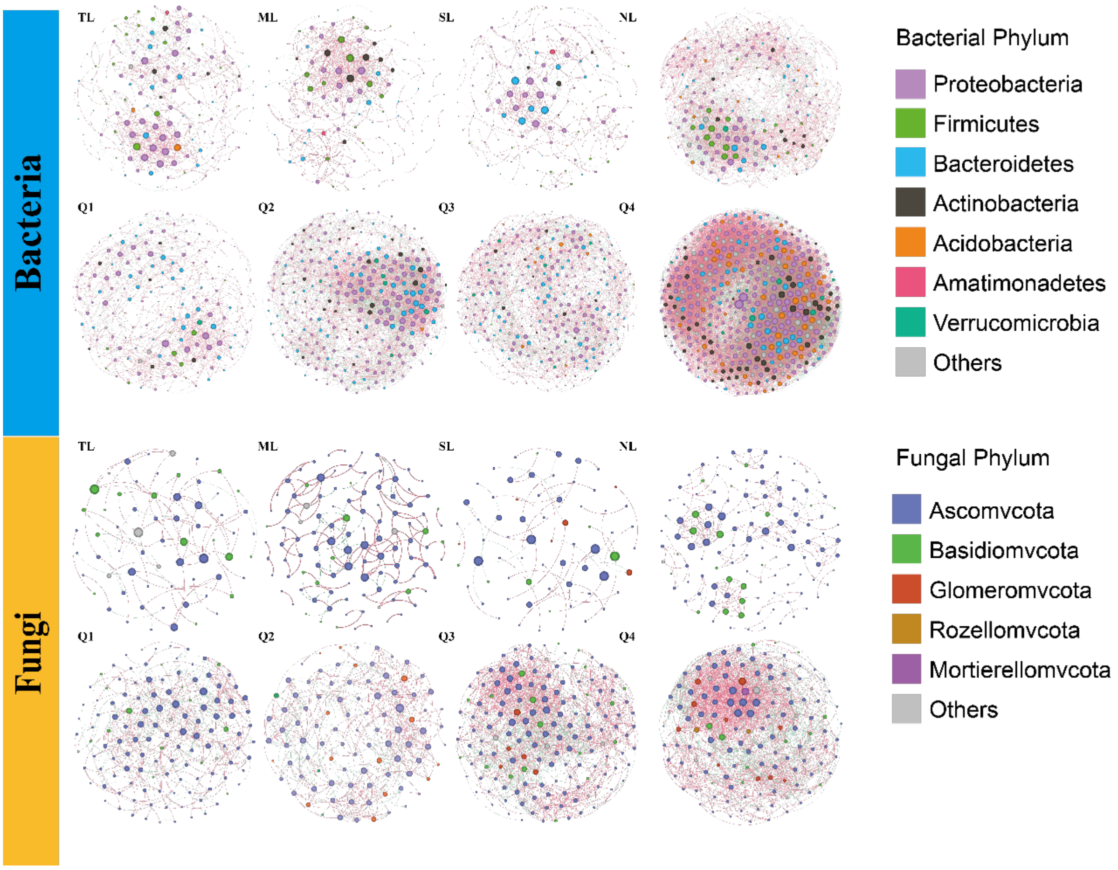
Bacteria
Fungi
TL
ML
SL
NL
Q1
Q2
Q3
Q4
Bacterial Phylum
Proteobacteria
Firmicutes
Bacteroidetes
Actinobacteria
Acidobacteria
Amatimonadetes
Verrucomicrobia
Others
Fungal Phylum
Ascomvcota
Basidiomvcota
Glomeromvcota
Rozellomvcota
Mortierellomvcota
Others

Figure S4

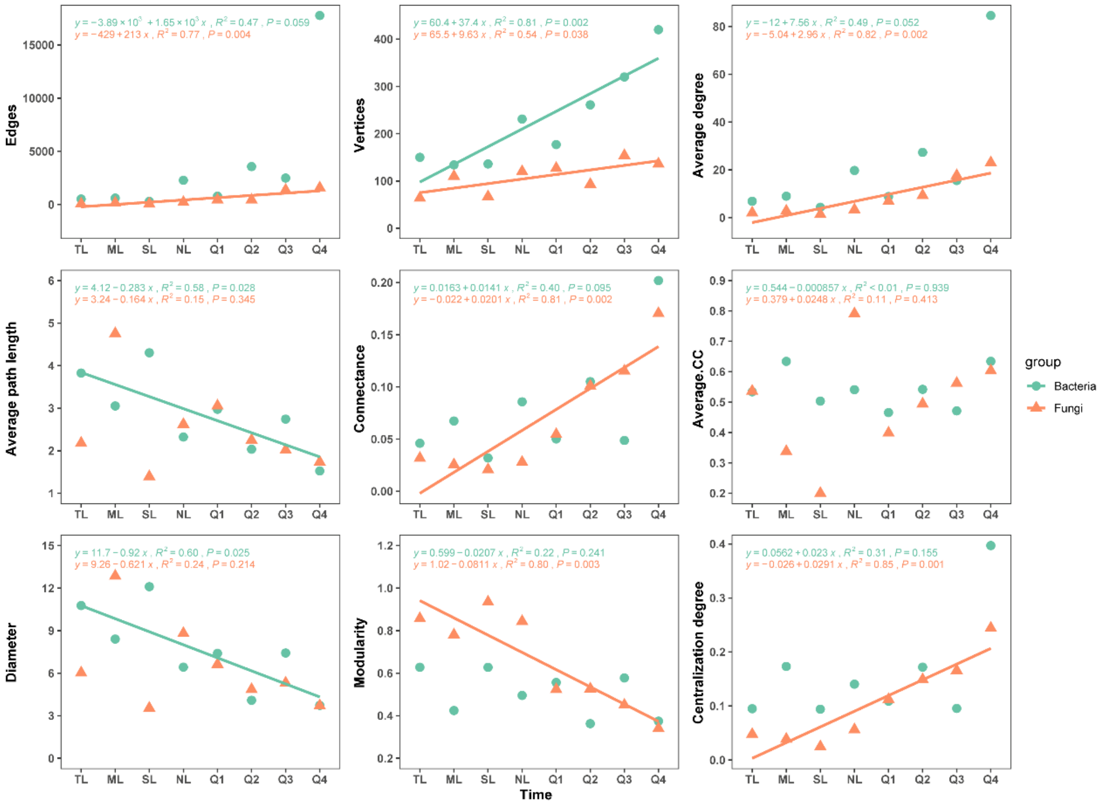

y = −3.89 × 10³ + 1.65 × 10³ x , R² = 0.47 , P = 0.059
y = −429 + 213 x , R² = 0.77 , P = 0.004
Edges
y = 60.4 + 37.4 x , R² = 0.81 , P = 0.002
y = 65.5 + 9.63 x , R² = 0.54 , P = 0.038
Vertices
y = −12 + 7.56 x , R² = 0.49 , P = 0.052
y = −5.04 + 2.96 x , R² = 0.82 , P = 0.002
Average degree
y = 4.12 − 0.283 x , R² = 0.58 , P = 0.028
y = 3.24 − 0.164 x , R² = 0.15 , P = 0.345
Average path length
y = 0.0163 + 0.0141 x , R² = 0.40 , P = 0.095
y = −0.022 + 0.0201 x , R² = 0.81 , P = 0.002
Connectance
y = 0.544 − 0.000857 x , R² < 0.01 , P = 0.939
y = 0.379 + 0.0248 x , R² = 0.11 , P = 0.413
Average.CC
group
Bacteria
Fungi
y = 11.7 − 0.92 x , R² = 0.60 , P = 0.025
y = 9.26 − 0.621 x , R² = 0.24 , P = 0.214
Diameter
y = 0.599 − 0.0207 x , R² = 0.22 , P = 0.241
y = 1.02 − 0.0811 x , R² = 0.80 , P = 0.003
Modularity
y = 0.0562 + 0.023 x , R² = 0.31 , P = 0.155
y = −0.026 + 0.0291 x , R² = 0.85 , P = 0.001
Centralization degree
TL ML SL NL Q1 Q2 Q3 Q4
Time

Figure S5

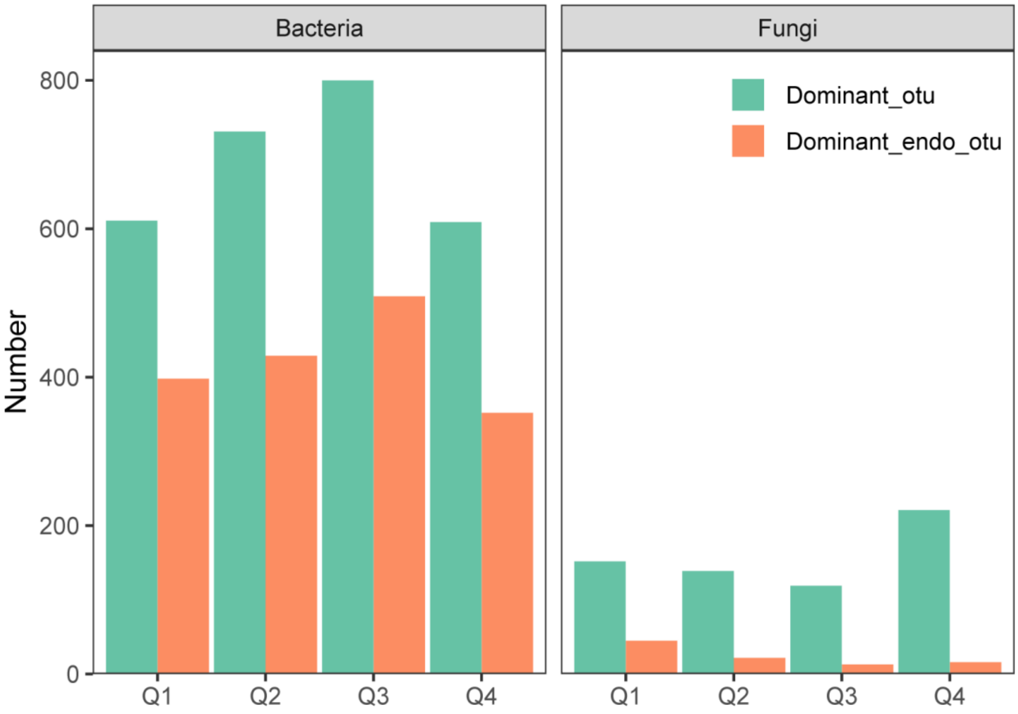

Bacteria
Fungi
Dominant_otu
Dominant_endo_otu
Number
800
600
400
200
0
Q1
Q2
Q3
Q4
Q1
Q2
Q3
Q4

Figure S6

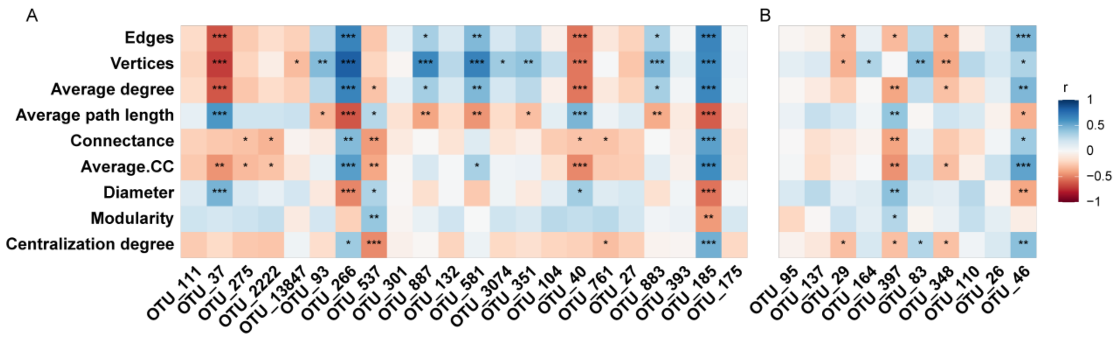
A
B
Edges
Vertices
Average degree
Average path length
Connectance
Average.CC
Diameter
Modularity
Centralization degree
OTU_111
OTU_37
OTU_275
OTU_2222
OTU_13847
OTU_93
OTU_266
OTU_537
OTU_301
OTU_887
OTU_132
OTU_581
OTU_3074
OTU_351
OTU_104
OTU_40
OTU_761
OTU_27
OTU_883
OTU_393
OTU_185
OTU_175
OTU_95
OTU_137
OTU_29
OTU_164
OTU_397
OTU_83
OTU_348
OTU_110
OTU_26
OTU_46
r
1
0.5
0
−0.5
−1

Figure S7

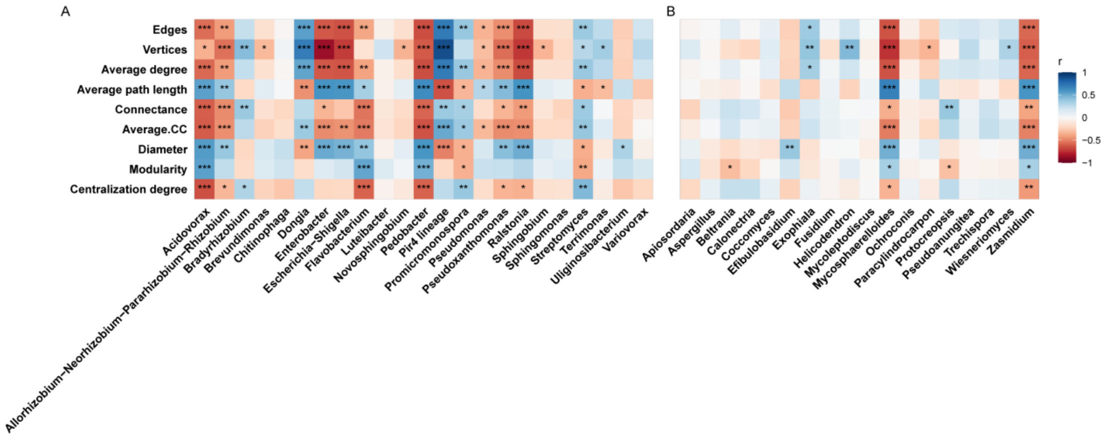
A
B
Edges
Vertices
Average degree
Average path length
Connectance
Average.CC
Diameter
Modularity
Centralization degree
Acidovorax
Allorhizobium−Neorhizobium−Pararhizobium−Rhizobium
Bradyrhizobium
Brevundimonas
Chitinophaga
Dongia
Enterobacter
Escherichia−Shigella
Flavobacterium
Luteibacter
Novosphingobium
Pedobacter
Pir4 lineage
Promicromonospora
Pseudomonas
Pseudoxanthomonas
Ralstonia
Sphingobium
Sphingomonas
Streptomyces
Terrimonas
Uliginosibacterium
Variovorax
Apiosordaria
Aspergillus
Beltrania
Calonectria
Coccomyces
Efibulobasidium
Exophiala
Fusidium
Helicodendron
Mycoleptodiscus
Mycosphaerelloides
Ochroconis
Paracylindrocarpon
Protocreopsis
Pseudoanungitea
Trechispora
Wiesneriomyces
Zasmidium
r
1
0.5
0
−0.5
−1

Figure S8

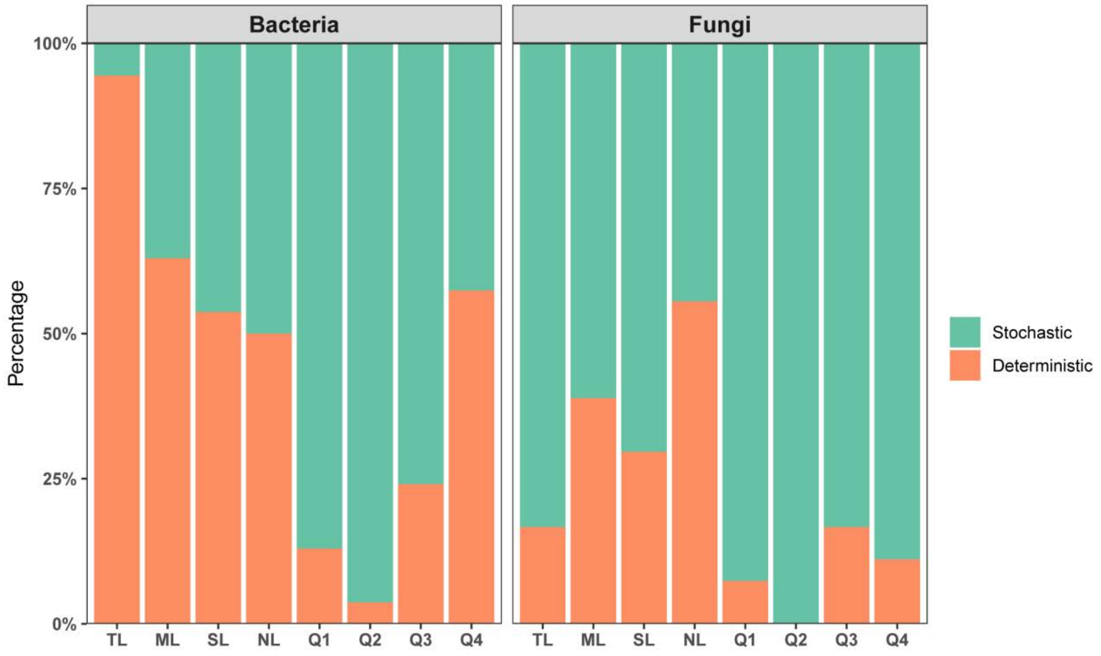
Bacteria
Fungi
100%
75%
50%
25%
0%
Percentage
TL
ML
SL
NL
Q1
Q2
Q3
Q4
Stochastic
Deterministic

Figure S9

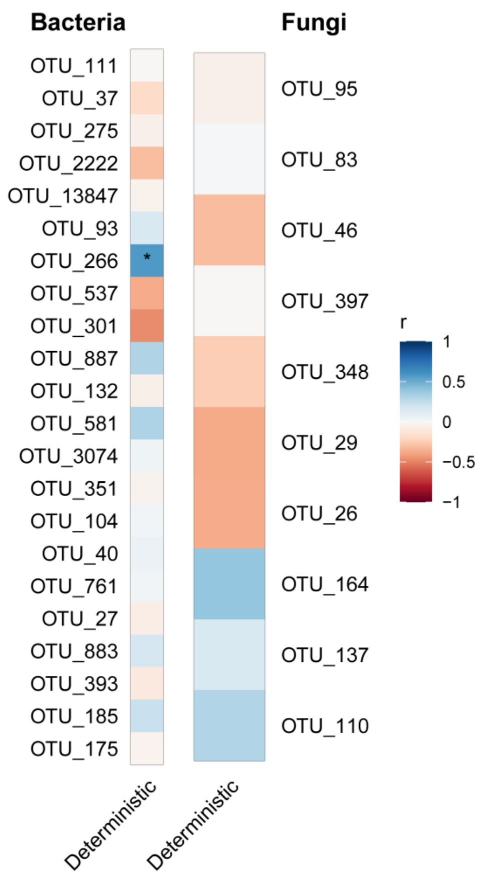

Bacteria
Fungi
OTU_111
OTU_37
OTU_275
OTU_2222
OTU_13847
OTU_93
OTU_266
*
OTU_537
OTU_301
OTU_887
OTU_132
OTU_581
OTU_3074
OTU_351
OTU_104
OTU_40
OTU_761
OTU_27
OTU_883
OTU_393
OTU_185
OTU_175
OTU_95
OTU_83
OTU_46
OTU_397
OTU_348
OTU_29
OTU_26
OTU_164
OTU_137
OTU_110
r
1
0.5
0
−0.5
−1
Deterministic
Deterministic

Figure S10

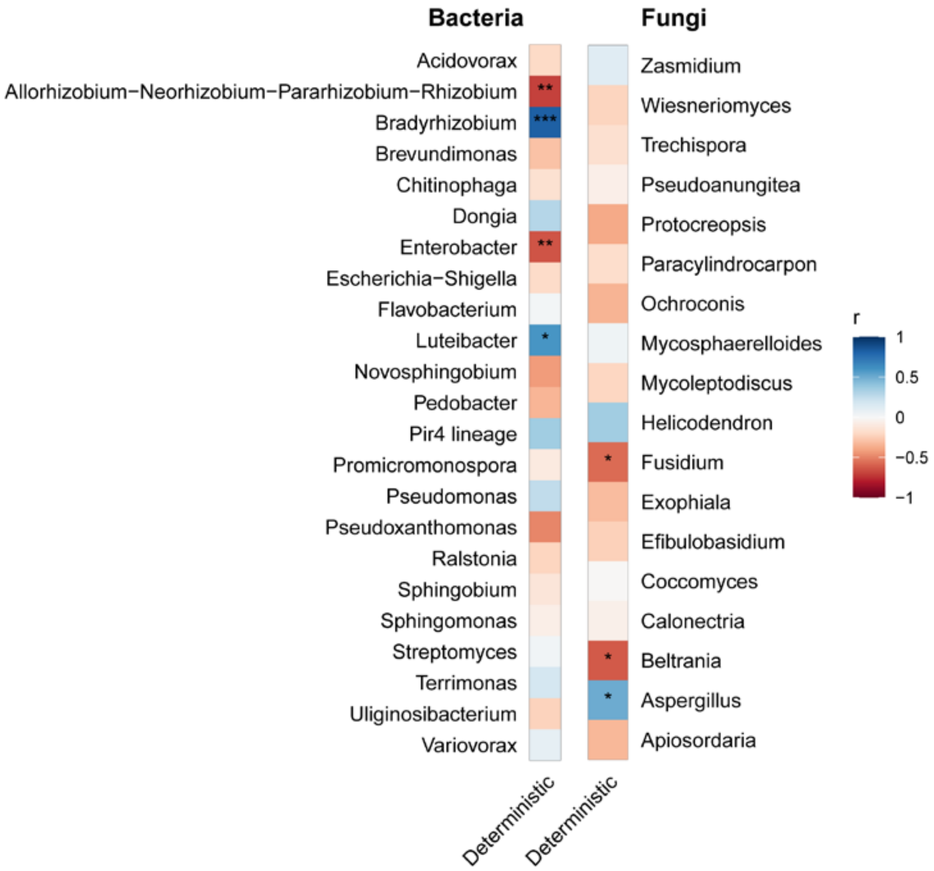
Bacteria
Acidovorax
Allorhizobium−Neorhizobium−Pararhizobium−Rhizobium
Bradyrhizobium
Brevundimonas
Chitinophaga
Dongia
Enterobacter
Escherichia−Shigella
Flavobacterium
Luteibacter
Novosphingobium
Pedobacter
Pir4 lineage
Promicromonospora
Pseudomonas
Pseudoxanthomonas
Ralstonia
Sphingobium
Sphingomonas
Streptomyces
Terrimonas
Uliginosibacterium
Variovorax
Fungi
Zasmidium
Wiesneriomyces
Trechispora
Pseudoanungitea
Protocreopsis
Paracylindrocarpon
Ochroconis
Mycosphaerelloides
Mycoleptodiscus
Helicodendron
Fusidium
Exophiala
Efibulobasidium
Coccomyces
Calonectria
Beltrania
Aspergillus
Apiosordaria
Deterministic
Deterministic
r
1
0.5
0
−0.5
−1

Figure S11

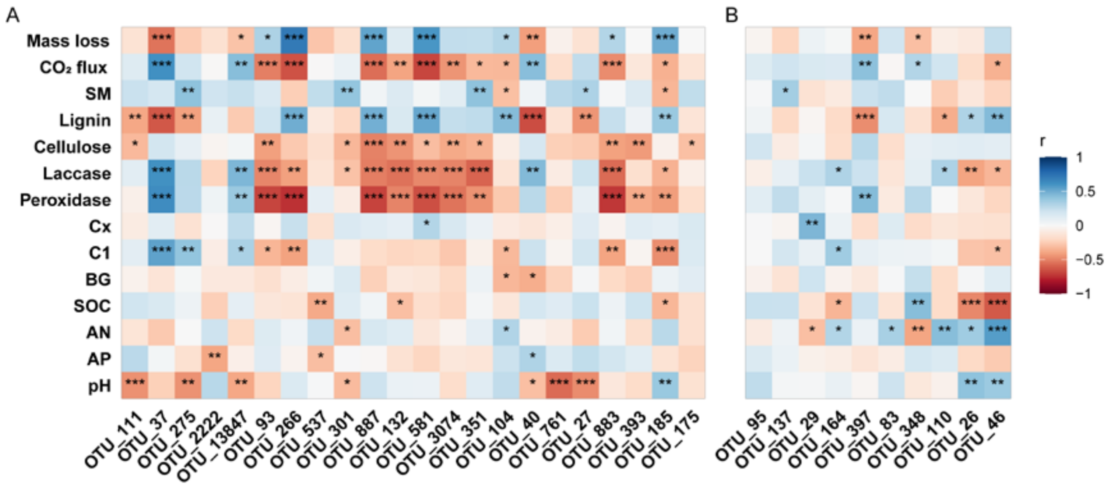
A
B
Mass loss
CO₂ flux
SM
Lignin
Cellulose
Laccase
Peroxidase
Cx
C1
BG
SOC
AN
AP
pH
OTU_111
OTU_37
OTU_275
OTU_2222
OTU_13847
OTU_93
OTU_266
OTU_537
OTU_301
OTU_887
OTU_132
OTU_581
OTU_3074
OTU_351
OTU_104
OTU_40
OTU_761
OTU_27
OTU_883
OTU_393
OTU_185
OTU_175
OTU_95
OTU_137
OTU_29
OTU_164
OTU_397
OTU_83
OTU_348
OTU_110
OTU_26
OTU_46
r
1
0.5
0
−0.5
−1

Figure S12

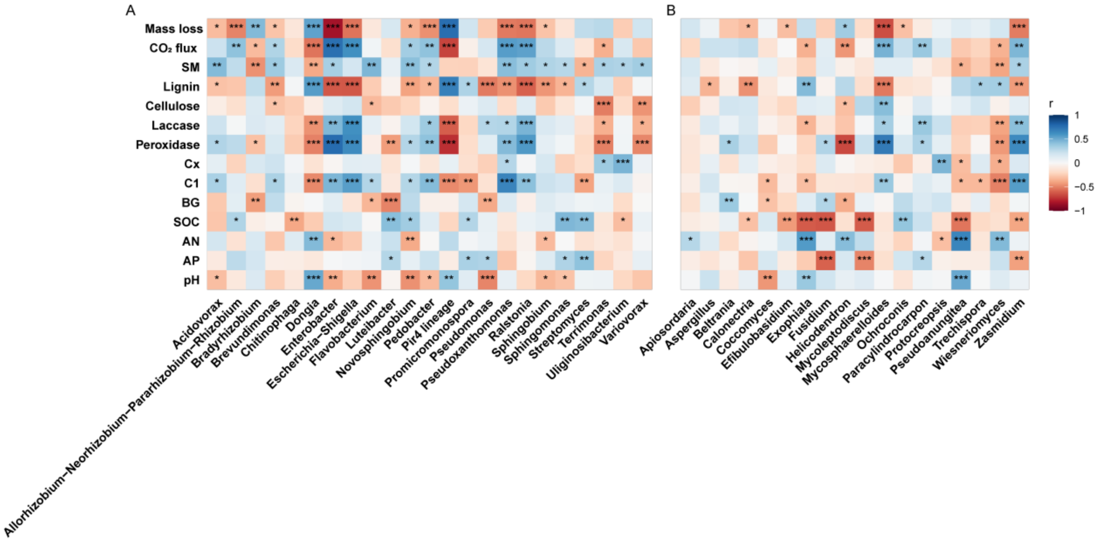
A
B
Mass loss
CO₂ flux
SM
Lignin
Cellulose
Laccase
Peroxidase
Cx
C1
BG
SOC
AN
AP
pH
Acidovorax
Allorhizobium−Neorhizobium−Pararhizobium−Rhizobium
Bradyrhizobium
Brevundimonas
Chitinophaga
Dongia
Enterobacter
Escherichia−Shigella
Flavobacterium
Luteibacter
Novosphingobium
Pedobacter
Pir4 lineage
Promicromonospora
Pseudomonas
Pseudoxanthomonas
Ralstonia
Sphingobium
Sphingomonas
Streptomyces
Terrimonas
Uliginosibacterium
Variovorax
Apiosordaria
Aspergillus
Beltrania
Calonectria
Coccomyces
Efibulobasidium
Exophiala
Fusidium
Helicodendron
Mycoleptodiscus
Mycosphaerelloides
Ochroconis
Paracylindrocarpon
Protocreopsis
Pseudoanungitea
Trechispora
Wiesneriomyces
Zasmidium
r
1
0.5
0
−0.5
−1

Figure S13
