# Supplementary material for: Tracing microbial community across endophyte-to-saprotroph continuum of Cinnamomum camphora (L.) Presl leaves considering priority effect of endophyte on litter decomposition
Source: Front Microbiol. 2025 Jan 15;15:1518569. doi: 10.3389/fmicb.2024.1518569 (PMC11774851; doi:10.3389/fmicb.2024.1518569)
Supplement: Supplementary file 2 [file Table_1.docx]

**Tracing microbial community across endophyte-to-saprotroph continuum of *Cinnamomum camphora* leaves considering priority effect of endophyte on litter decomposition**

Jiamin Xiao^1^, Zaihua He^1^, Xingbing He^1^, Yonghui Lin^1,*^, Xiangshi Kong^2^

^1^College of Biology and Environmental Sciences, Jishou University, Jishou 416000, China

^2^College of Tourism and Management Engineering, Jishou University, Zhangjiajie 427000, China

^*^ Corresponding author:

Yonghui Lin

E–mail: [linyonghui@jsu.edu.cn](mailto:linyonghui@jsu.edu.cn)

Address: College of Biology and Environmental Sciences, Jishou University, Jishou, Hunan, 416000, China.

Table S1 Sampling dates and information of living leaves and leaf litters of *C. camphora* forest.

| Site | Longitude | Latitude | SWC(%) | pH | SOM(mg/kg) | AP(mg/kg) | AN(mg/kg) | TL | ML | SL | NL | Q1 | Q2 | Q3 | Q4 |
| --- | --- | --- | --- | --- | --- | --- | --- | --- | --- | --- | --- | --- | --- | --- | --- |
| HS | 109°43'E | 28°17'N | 35.27±1.45(c) | 5.86±0.01(c) | 7.82±0.20(c) | 28.51±0.32(a) | 33.88±0.32(a) | 2020.02.22 | 2020.07.05 | 2020.10.06 | 2020.10.07 | 2020.11.08 | 2021.01.08 | 2021.04.08 | 2021.10.08 |
| HGS | 109°44'E | 28°19'N | 29.36±1.45(b) | 6.18±0.01(d) | 4.20±0.06(a) | 23.80±0.32(a) | 27.55±3.22(b) | 2020.02.23 | 2020.07.06 | 2020.10.08 | 2020.10.09 | 2020.11.09 | 2021.01.09 | 2021.04.09 | 2021.10.09 |
| XN | 101°46'E | 26°24'N | 23.82±1.16(a) | 5.61±0.01(a) | 8.68±0.12(d) | 64.59±1.91(c) | 37.04±2.79(c) | 2020.02.26 | 2020.07.13 | 2020.10.12 | 2020.10.13 | 2020.11.13 | 2021.01.13 | 2021.04.13 | 2021.10.13 |
| ZJJ | 110°27'E | 29°8'N | 36.32±1.21(c) | 5.831±0.03(b) | 7.65±0.08(b) | 41.54±4.65(b) | 28.58±0.73(c) | 2020.02.29 | 2020.07.16 | 2020.10.17 | 2020.10.18 | 2020.11.17 | 2021.01.17 | 2021.04.17 | 2021.10.17 |

The values of soil physicochemical properties are presented as Mean ± SE. Different lowercase letters after each set of numbers indicate a significant difference (*p* < 0.05). SWC: Soil Water Content; SOM: Soil Organic Matter; AP: Available Phosphorus; AN: Available Nitrogen; TL (tender leaves), ML (mature leaves), SL (senescent leaves) and NL (newly-fallen leaves) are developmental phases of living leaves, and the Q1 (initial), Q2 (early), Q3 (middle) and Q4 (late) are the stages of leaf litter decomposition.

Table S2 The parameters of co-occurrence networks for bacteria and fungi of each stage along endophyte-to-saprotroph continuum of *C*. *camphora* leaves

| Taxa | Stage | Num.edge (L) | Num.vertices (n) | Average.degree (Average K) | Average.path.  length | Connectance (edge_density) | Mean.clustering.coefficient (Average.CC) | Diameter | Modularity.net | Centralization.degree |
| --- | --- | --- | --- | --- | --- | --- | --- | --- | --- | --- |
| Bacteria | TL | 514 | 150 | 6.85 | 3.83 | 0.05 | 0.53 | 10.77 | 0.63 | 0.09 |
|  | ML | 600 | 134 | 8.96 | 3.05 | 0.07 | 0.63 | 8.41 | 0.42 | 0.17 |
|  | SL | 293 | 136 | 4.31 | 4.30 | 0.03 | 0.50 | 12.10 | 0.63 | 0.09 |
|  | NL | 2278 | 231 | 19.72 | 2.32 | 0.09 | 0.54 | 6.42 | 0.50 | 0.14 |
|  | Q1 | 782 | 177 | 8.84 | 2.97 | 0.05 | 0.47 | 7.40 | 0.56 | 0.11 |
|  | Q2 | 3565 | 261 | 27.32 | 2.04 | 0.11 | 0.54 | 4.09 | 0.36 | 0.17 |
|  | Q3 | 2486 | 320 | 15.54 | 2.74 | 0.05 | 0.47 | 7.42 | 0.58 | 0.10 |
|  | Q4 | 17769 | 420 | 84.61 | 1.52 | 0.20 | 0.63 | 3.73 | 0.37 | 0.40 |
| Fungi | TL | 64 | 64 | 2.00 | 2.18 | 0.03 | 0.54 | 6.03 | 0.86 | 0.05 |
|  | ML | 153 | 110 | 2.78 | 4.75 | 0.03 | 0.34 | 12.85 | 0.78 | 0.04 |
|  | SL | 46 | 67 | 1.37 | 1.39 | 0.02 | 0.20 | 3.54 | 0.93 | 0.02 |
|  | NL | 199 | 120 | 3.32 | 2.62 | 0.03 | 0.79 | 8.81 | 0.84 | 0.06 |
|  | Q1 | 436 | 127 | 6.87 | 3.06 | 0.05 | 0.40 | 6.63 | 0.52 | 0.11 |
|  | Q2 | 432 | 93 | 9.29 | 2.25 | 0.10 | 0.49 | 4.86 | 0.53 | 0.15 |
|  | Q3 | 1358 | 154 | 17.64 | 2.02 | 0.12 | 0.56 | 5.31 | 0.45 | 0.17 |
|  | Q4 | 1565 | 136 | 23.01 | 1.73 | 0.17 | 0.60 | 3.71 | 0.34 | 0.24 |

(TL (tender leaves), ML (mature leaves), SL (senescent leaves) and NL (newly-fallen leaves) are developmental phases of living leaves, and the Q1 (initial), Q2 (early), Q3 (middle) and Q4 (late) are the stages of leaf litter decomposition. The content inside the parentheses is an abbreviation for the preceding text).

Table S3 A classification list of keystone taxa at different taxonomic levels along the endophyte-to-saprotroph continuum of *C*. *camphora* leaves

|  | Keystone taxa | Kingdom | Phylum | Class | Order | Family |
| --- | --- | --- | --- | --- | --- | --- |
| TL | BOTU_369 | Bacteria | Proteobacteria | Alphaproteobacteria | Caulobacterales | Caulobacteraceae |
| TL | BOTU_753 | Bacteria | Proteobacteria | Alphaproteobacteria | Rhizobiales | Rhizobiaceae |
| SL | BOTU_1021 | Bacteria | Proteobacteria | Alphaproteobacteria | Rhizobiales | Beijerinckiaceae |
| SL | BOTU_21 | Bacteria | Proteobacteria | Alphaproteobacteria | Caulobacterales | Caulobacteraceae |
| SL | BOTU_273 | Bacteria | Proteobacteria | Alphaproteobacteria | Sphingomonadales | Sphingomonadaceae |
| NL | BOTU_150 | Bacteria | Proteobacteria | Alphaproteobacteria | Rhizobiales | Beijerinckiaceae |
| NL | BOTU_171 | Bacteria | Bacteroidetes | Bacteroidia | Bacteroidales | Muribaculaceae |
| Q1 | BOTU_111 | Bacteria | Proteobacteria | Alphaproteobacteria | Caulobacterales | Caulobacteraceae |
| Q1 | BOTU_2222 | Bacteria | Proteobacteria | Gammaproteobacteria | Xanthomonadales | Xanthomonadaceae |
| Q1 | BOTU_275 | Bacteria | Bacteroidetes | Bacteroidia | Flavobacteriales | Flavobacteriaceae |
| Q1 | BOTU_37 | Bacteria | Proteobacteria | Gammaproteobacteria | Enterobacteriales | Enterobacteriaceae |
| Q2 | BOTU_13847 | Bacteria | Proteobacteria | Gammaproteobacteria | Enterobacteriales | Enterobacteriaceae |
| Q2 | BOTU_93 | Bacteria | Verrucomicrobia | Verrucomicrobiae | Methylacidiphilales | Methylacidiphilaceae |
| Q3 | BOTU_104 | Archaea | Thaumarchaeota | Nitrososphaeria | Nitrososphaerales | Nitrososphaeraceae |
| Q3 | BOTU_132 | Bacteria | Proteobacteria | Gammaproteobacteria | R7C24 | Unclassified |
| Q3 | BOTU_266 | Bacteria | Proteobacteria | Alphaproteobacteria | Rhizobiales | Xanthobacteraceae |
| Q3 | BOTU_301 | Bacteria | Proteobacteria | Alphaproteobacteria | Sphingomonadales | Sphingomonadaceae |
| Q3 | BOTU_3074 | Bacteria | Bacteroidetes | Bacteroidia | Cytophagales | Microscillaceae |
| Q3 | BOTU_351 | Bacteria | Proteobacteria | Alphaproteobacteria | Sphingomonadales | Sphingomonadaceae |
| Q3 | BOTU_40 | Bacteria | Proteobacteria | Gammaproteobacteria | Pseudomonadales | Pseudomonadaceae |
| Q3 | BOTU_537 | Bacteria | Bacteroidetes | Bacteroidia | Chitinophagales | Chitinophagaceae |
| Q3 | BOTU_581 | Bacteria | Planctomycetes | Planctomycetacia | Gemmatales | Gemmataceae |
| Q3 | BOTU_761 | Bacteria | Proteobacteria | Gammaproteobacteria | Betaproteobacteriales | Burkholderiaceae |
| Q3 | BOTU_887 | Bacteria | Proteobacteria | Gammaproteobacteria | Pseudomonadales | Moraxellaceae |
| Q4 | BOTU_175 | Bacteria | Verrucomicrobia | Verrucomicrobiae | Verrucomicrobiales | Rubritaleaceae |
| Q4 | BOTU_185 | Bacteria | Proteobacteria | Alphaproteobacteria | Dongiales | Dongiaceae |
| Q4 | BOTU_27 | Bacteria | Proteobacteria | Gammaproteobacteria | Xanthomonadales | Xanthomonadaceae |
| Q4 | BOTU_393 | Bacteria | Proteobacteria | Alphaproteobacteria | Sphingomonadales | Sphingomonadaceae |
| Q4 | BOTU_761 | Bacteria | Proteobacteria | Gammaproteobacteria | Betaproteobacteriales | Burkholderiaceae |
| Q4 | BOTU_883 | Bacteria | Proteobacteria | Alphaproteobacteria | Rhizobiales | Xanthobacteraceae |
| ML | FOTU_51 | Fungi | Ascomycota | Dothideomycetes | Capnodiales | Unclassified |
| ML | FOTU_60 | Fungi | Ascomycota | Dothideomycetes | Capnodiales | Mycosphaerellaceae |
| ML | FOTU_797 | Fungi | Ascomycota | Eurotiomycetes | Onygenales | Onygenales_fam_Incertae_sedis |
| Q1 | FOTU_137 | Fungi | Ascomycota | Dothideomycetes | Capnodiales | Mycosphaerellaceae |
| Q1 | FOTU_153 | Fungi | Ascomycota | Sordariomycetes | Hypocreales | Hypocreaceae |
| Q1 | FOTU_164 | Fungi | Ascomycota | Sordariomycetes | Sordariales | Unclassified |
| Q1 | FOTU_29 | Fungi | Ascomycota | Sordariomycetes | Hypocreales | Bionectriaceae |
| Q1 | FOTU_397 | Fungi | Ascomycota | Dothideomycetes | Capnodiales | Dissoconiaceae |
| Q1 | FOTU_95 | Fungi | Ascomycota | Sordariomycetes | Hypocreales | Hypocreales_fam_Incertae_sedis |
| Q2 | FOTU_255 | Fungi | Ascomycota | Dothideomycetes | Pleosporales | Pleosporales_fam_Incertae_sedis |
| Q2 | FOTU_348 | Fungi | Ascomycota | Sordariomycetes | Diaporthales | Diaporthaceae |
| Q2 | FOTU_83 | Fungi | Unclassified | Unclassified | Unclassified | Unclassified |
| Q3 | FOTU_195 | Fungi | Ascomycota | Pezizomycotina_cls_Incertae_sedis | Pezizomycotina_ord_Incertae_sedis | Pezizomycotina_fam_Incertae_sedis |
| Q3 | FOTU_2524 | Fungi | Ascomycota | Dothideomycetes | Venturiales | Sympoventuriaceae |
| Q4 | FOTU_110 | Fungi | Unclassified | Unclassified | Unclassified | Unclassified |
| Q4 | FOTU_253 | Fungi | Ascomycota | Leotiomycetes | Helotiales | Unclassified |
| Q4 | FOTU_26 | Fungi | Basidiomycota | Agaricomycetes | Sebacinales | Sebacinaceae |
| Q4 | FOTU_28 | Fungi | Ascomycota | Leotiomycetes | Helotiales | Helotiales_fam_Incertae_sedis |
| Q4 | FOTU_302 | Fungi | Ascomycota | Sordariomycetes | Xylariales | Unclassified |
| Q4 | FOTU_46 | Fungi | Ascomycota | Eurotiomycetes | Chaetothyriales | Herpotrichiellaceae |

(TL (tender leaves), ML (mature leaves), SL (senescent leaves) and NL (newly-fallen leaves) are developmental phases of living leaves, and the Q1 (initial), Q2 (early), Q3 (middle) and Q4 (late) are the stages of leaf litter decomposition.)
